# Long wavelength-sensing cones of zebrafish retina exhibit multiple layers of transcriptional heterogeneity

**DOI:** 10.3389/fncel.2023.1214084

**Published:** 2023-07-14

**Authors:** Ashley A. Farre, Chi Sun, Margaret R. Starostik, Samuel S. Hunter, Milton A. English, Audrey Duncan, Abirami Santhanam, Eyad Shihabeddin, John O’Brien, Anand Swaroop, Deborah L. Stenkamp

**Affiliations:** ^1^Department of Biological Sciences, University of Idaho, Moscow, ID, United States; ^2^Neurobiology-Neurodegeneration and Repair Laboratory, National Eye Institute, National Institutes of Health, Bethesda, MD, United States; ^3^Department of Vision Science, University of Houston College of Optometry, Houston, TX, United States; ^4^MD Anderson Cancer Center UTHealth Houston Graduate School of Biomedical Sciences, Houston, TX, United States

**Keywords:** cone, photoreceptor, zebrafish, retina, RNA-Seq, scRNA-Seq, opsin, transducin

## Abstract

**Introduction:**

Understanding how photoreceptor genes are regulated is important for investigating retinal development and disease. While much is known about gene regulation in cones, the mechanism by which tandemly-replicated opsins, such as human long wavelength-sensitive and middle wavelength-sensitive opsins, are differentially regulated remains elusive. In this study, we aimed to further our understanding of transcriptional heterogeneity in cones that express tandemly-replicated opsins and the regulation of such differential expression using zebrafish, which express the tandemly-replicated opsins *lws1* and *lws2*.

**Methods:**

We performed bulk and single cell RNA-Seq of LWS1 and LWS2 cones, evaluated expression patterns of selected genes of interest using multiplex fluorescence *in situ* hybridization, and used exogenous thyroid hormone (TH) treatments to test selected genes for potential control by thyroid hormone: a potent, endogenous regulator of *lws1* and *lws2* expression.

**Results:**

Our studies indicate that additional transcriptional differences beyond opsin expression exist between LWS1 and LWS2 cones. Bulk RNA-Seq results showed 95 transcripts enriched in LWS1 cones and 186 transcripts enriched in LWS2 cones (FC > 2, FDR < 0.05). *In situ* hybridization results also reveal underlying heterogeneity within the *lws1*- and *lws2*-expressing populations. This heterogeneity is evident in cones of mature zebrafish, and further heterogeneity is revealed in transcriptional responses to TH treatments.

**Discussion:**

We found some evidence of coordinate regulation of *lws* opsins and other genes by exogenous TH in LWS1 vs. LWS2 cones, as well as evidence of gene regulation not mediated by TH. The transcriptional differences between LWS1 and LWS2 cones are likely controlled by multiple signals, including TH.

## 1. Introduction

Cone photoreceptors of vertebrates express specific opsins that maximally detect specific wavelengths of light. The presence of multiple types of cones that express opsin proteins with different peak spectral tuning allows for color vision. Humans have three different types of cones (red-, green-, and blue-sensitive) that each express a specific cone opsin: long wavelength-sensitive (LWS), middle wavelength sensitive (MWS), and short wavelength sensitive (SWS) opsins, respectively (Nathans, 1999). The genes encoding the human LWS and MWS opsins are arranged in tandem on the X chromosome (Vollrath et al., 1988), and the mechanism by which they are regulated remains largely unknown, although several models have been suggested (Wang et al., 1999; Peng and Chen, 2011; Hussey et al., 2022). Mutations in these opsin genes have been associated with multiple visual disorders including color vision deficiencies, high myopia, X-linked cone dysfunction, and X-linked cone dystrophy (Winderickx et al., 1992; Ayyagari et al., 1999; Young et al., 2004; Michaelides et al., 2005; Gardner et al., 2010).

Although mice remain the predominant model system in molecular and cellular biology, and have been instrumental for vision research, they lack tandemly replicated opsin genes. The only mammals known to share this gene structure with humans are other primates and bats (Hofmann and Carleton, 2009), for which genetic and other experimental manipulations that would be useful for the study of gene expression are severely limited and/or practically difficult. The zebrafish, however, is a vertebrate model organism that does possess tandemly replicated opsins (Chinen et al., 2003), and for which many genetic tools have been developed (Niklaus and Neuhauss, 2017). Further, the tandemly duplicated zebrafish *lws* opsin genes and the human *LWS/MWS* opsin genes evolved from a common ancestral long wavelength-sensing opsin gene. Therefore, the zebrafish provides an excellent opportunity to study gene expression in cones that express tandemly replicated opsins. Previous research using the zebrafish and other model organisms has shown that thyroid hormone (TH) is essential in determining cone subtype identity and patterning (Ng et al., 2001; Roberts et al., 2006; Forrest and Swaroop, 2012; Boyes et al., 2018; Mackin et al., 2019). This appears to be true for humans as well, as shown for stem cell-derived retinal organoids (Eldred et al., 2018). Recent studies from our lab have also demonstrated that TH can promote the expression of some tandemly replicated opsins over others, a conserved phenomenon for both of the tandemly replicated cone opsin arrays in zebrafish. In both the *lws* and *rh2* (middle wavelength-sensitive in non-mammalian vertebrates) arrays, TH promoted the expression of the long wavelength-shifted member(s) of the array at the expense of the more short-wavelength-shifted member(s) (Mackin et al., 2019). Further, treatment with exogenous TH can cause larval cones expressing one member of the *lws1/lws2* tandem array to “switch” and begin expressing another member of the array (Mackin et al., 2019).

Larval, juvenile, and adult zebrafish have characteristic *lws* opsin spatiotemporal expression patterns. In larval zebrafish, *lws2* expression begins at 40 h post-fertilization (hpf) in the central and dorsal retina while *lws1* expression begins at approximately 5 days post-fertilization (dpf) in the ventral region. In adults, ventral and nasal LWS cones express *lws1* while central and dorsal LWS cones express *lws2* (Takechi and Kawamura, 2005; Tsujimura et al., 2010). Larval and juvenile zebrafish made experimentally athyroid display abnormal *lws1* vs. *lws2* expression patterns, supporting endogenous roles for TH in the regulation of their differential expression (Mackin et al., 2019).

In this study, we aimed to further our understanding of the cones that express tandemly replicated opsins and the regulation of opsin expression by performing bulk and single cell RNA-Seq of LWS1 and LWS2 cones. We then investigated spatial patterning of several transcripts found to be differential expressed, in adult whole retina, and in larval retinas with or without exogenous thyroid hormone treatment. Our goals were to determine whether LWS1 vs. LWS2 cone subtypes exhibit transcriptional differences beyond opsin expression, to probe potential mechanisms for opsin switching and differential tandemly replicated opsin expression, and to investigate the role of TH in regulating differences between cone subtypes that express tandemly replicated opsins. Our studies indicate that additional transcriptional differences exist between these cone subtypes beyond opsin expression, and reveal underlying heterogeneity within the *lws1*- and *lws2*-expressing populations. This heterogeneity is evident in cones of mature and larval zebrafish, and further heterogeneity is revealed in transcriptional responses to TH treatments.

## 2. Materials and methods

### 2.1. Animals

Zebrafish were propagated and maintained as described (Westerfield, 2007), on recirculating, monitored, and filtered system water, on a 14:10 light/dark cycle, at 28.5°C. Procedures involving animals were approved by the Animal Care and Use Committees of the University of Idaho and of the University of Texas Health Science Center at Houston. Wild-type (WT) zebrafish were of a strain originally provided by Scientific Hatcheries (now Aquatica Tropicals, Plant City, FL) and AB (RRID:ZIRC_ZL1) from the Zebrafish International Resource Center at the University of Oregon. The *lws:PAC(H)* transgenic line harbors a PAC clone that encompasses the *lws* locus, modified such that a GFP-polyA sequence, inserted after the *lws1* promoter, reports expression of *lws1*, and an RFP (dsRedExpress)-polyA sequence, inserted after the *lws2* promoter, reports expression of *lws2* (Tsujimura et al., 2010). This line was the kind gift of Shoji Kawamura and the RIKEN international resource facility. The *thrb2:tdTomato* transgenic line expresses the tdTomato reporter under control of *the thyroid hormone receptor beta 2 promoter*, resulting in tdTomato in all adult LWS cones (Suzuki et al., 2013). This line was the kind gift of Rachel Wong. In this study larval (4 dpf) and adult (0.5–1.5 years; both sexes) zebrafish were used.

### 2.2. Retinal tissue dissociation and fluorescence-activated cell sorting (FACS)

Dissociation and FACS were carried out as previously reported (Sun et al., 2018a,b), for bulk RNA-Seq and for qPCR. In brief, adult *lws:PAC(H)* and *thrb2:tdTomato* zebrafish were collected near the time of light onset but maintained in the dark (dark-adapted), euthanized with MS-222, and retinal tissues dissected away from other ocular tissues including the RPE and collected into cold RNAse-free phosphate-buffered saline (PBS). Retinas were dissociated for 10 min at 37°C in a filtered buffer containing papain, trypsin, neutral protease, catalase, and superoxide dismutase. The reaction was quenched with heat-inactivated fetal bovine serum, and samples resuspended in DNAseI for 10 min at room temperature. Samples were pelleted and resuspended in RNAse-free PBS prior to FACS. *Lws:PAC(H)* samples were sorted with a SONY Cell Sorter SH800 based upon GFP and RFP fluorescence, and collected into TRIzol LS or lysis buffer from the Machery–Nagel RNA extraction kit (Sun et al., 2018b). *Thrb2:tdTomato* samples were sorted using the same instrument and conditions, but based upon tdTomato fluorescence intensity and the scatter characteristics (Sun et al., 2018b).

### 2.3. Bulk RNA-sequencing (bulk RNA-Seq)

The quality of isolated RNA was evaluated using the Bioanalyzer 2100 RNA 6000 Nano assay (Agilent Technologies). For samples with a RIN score greater than 8.0, sequencing libraries were constructed according to the manufacturer’s protocol using the TruSeq^®^ RNA Library Preparation Kit (Illumina) with 5 μg total RNA as input whereby ribosomal RNA was removed by poly-A selection using Illumina. Illumina sequencing adapters were ligated to each sample. Ligated fragments were then amplified for 12 cycles using primers incorporating unique dual index tags. Libraries were sequenced at the National Eye Institute (NEI) on the Illumina HiSeq 2500 platform, and raw sequencing reads were demultiplexed by NEI. Raw bulk RNA-Seq reads were trimmed for Illumina adapters and quality using Trimmomatic v0.36 (Bolger et al., 2014). Trimmed reads were aligned to the zebrafish reference genome GRCz11 using STAR v2.5.2a (Dobin et al., 2013) and Salmon v1.0.0 (Patro et al., 2017). Approximately 1–3 million reads were mapped per sample library (3 libraries per condition) to zebrafish reference genome GRCz11, with the exception of one *lws1*:GFP + library, which still provided minimal depth for downstream analyses, and so was included in subsequent analyses to increase statistical power. Additional quality control metrics were evaluated using FastQC v0.11.5^1^ and HTStream (Petersen et al., 2015). Data were imported into R (R Core Team, 2017)^2^ using tximport (Soneson et al., 2015). Differential expression (DE) analysis was then carried out using DESeq2 (Love et al., 2014). Log_2_FC, as well as a moderated Log_2_FC (to normalize for transcripts displaying very low levels of expression) were determined for *lws1*:GFP vs. *lws2*:RFP DE analyses. As additional strategies for identifying transcripts DE in LWS1 vs. LWS2 cones, DE analyses were also carried out for the entire LWS cone population (*thrb2*:tdTomato +) vs. *lws1*:GFP and vs. *lws2*:GFP. Gene ontology analyses were performed using gProfiler.

### 2.4. Retinal tissue dissociation and single-cell RNA-Seq (scRNA-Seq)

Single-cell library preparation and data analysis are fully described in Santhanam et al. (2022). In brief, two female and one male wild-type AB strain zebrafish, age 7 months, were euthanized with MS222 on ice and isolated retina-RPE preparations were pooled in a 3:1 mix of Leibovitz’s L-15 medium (Gibco) and Earle’s Balanced Salt Solution (EBSS; Gibco). Cells were dissociated with papain solution (50 U/mL; Worthington Biochemical) for 60 min at 28°C with gentle trituration. Digestion was halted by addition of 2x volume of 0.1% BSA in L15: EBSS medium, and cells were counted and tested for viability. Cell samples were submitted for 3′ scRNA library preparation and sequencing through the Baylor College of Medicine Single Cell Genomics Core facility. The single-cell library was prepared using a Chromium Next GEM Single Cell 3′ Reagent Kit v2 (10x Genomics, Pleasanton, CA, USA). The single-cell library was sequenced with Illumina HiSeq2500.

The single-cell library sequences were initially analyzed using the 10x Genomics CellRanger V2.1.1.0 pipeline. Sequences were aligned to the zebrafish reference genome GRCz11 using CellRanger count, and quality-checked using FASTQC V0.11.9. Over 93% of reads mapped successfully to the genome. The initial alignment and analysis were performed through the Texas Advanced Computing Center (TACC) Lonestar5 computing service. Subsequent analyses of aligned data used the Seurat V2.1.1 (Butler et al., 2018) package in R V3.6.1. The data were initially filtered to remove low-abundance genes (expressed in fewer than 10 cells), doublets and cells with >5% mitochondrial genes. The dataset contained between 200 and 4,000 genes per cell, and 13,551 cells were sequenced/analyzed. PCElbowPlots were performed and 20 principal components were used for downstream analysis of each dataset. PC1 to PC20 were used to construct nearest neighbor graphs in the PCA space followed by Louvain clustering and non-linear dimensional reduction by TSNE to visualize and explore the clusters. Expression levels are expressed in a base 2 log scale.

### 2.5. Thyroid hormone treatments

Stock solutions of tri-iodothyronine (T3) were prepared in DMSO (Sigma), and maintained at −20°C in the dark. Embryos were obtained from WT crosses, with the time of light onset considered the time of fertilization. 0.003% phenylthiourea (PTU) was added to system water at 10–12 h post-fertilization (hpf) to inhibit melanin synthesis [13]. Prior to T3 treatment, embryos were dechorionated using fine forceps, and the 1000X T3 stock solution was added to system water for a final concentration of 100 nM (DMSO final concentration was 0.1%). Controls were treated with 0.1% DMSO. Treatments took place from 48 to 96 hpf, and solutions were refreshed every 24 h (Mackin et al., 2019).

### 2.6. RNA extraction and quantitative RT-PCR (qPCR)

Total RNA from larval (4 dpf) zebrafish tissues was extracted using the Machery-Nagel kit, and then the Superscript III/IV (Invitrogen) was used to synthesize cDNA template with random primers. Gene-specific primer pairs for qPCR are provided in Supplementary Table 1. Amplification was performed on a StepOne Real-Time PCR system using SYBR Green or Power Track SYBR Green master mix (Applied Biosystems). Quantification of transcript abundance was relative to the reference transcript (β*-actin*), using the ddCT method. Graphing and statistics were performed in Excel. Sample groups were evaluated for normal distributions using the Shapiro–Wilk test. For comparisons showing normal distributions, *p*-values were calculated using Student’s *t*-test, and for comparisons not showing normal distributions, *p*-values were calculated using Mann–Whitney tests.

### 2.7. Hybridization chain reaction (HCR) *in situ* hybridization

Hybridization chain reaction procedures were carried out according to the manufacturer’s instructions (Molecular Instruments) (Choi et al., 2014). In brief, zebrafish tissues were fixed overnight in phosphate-buffered 4% paraformaldehyde at 4°C. Tissues were then washed in PBS, dehydrated in MeOH, and stored in MeOH at −20°C at least overnight. Tissues were rehydrated in a graded MeOH/PBS/0.1% Tween 20 series, permeabilized with proteinase K (larvae only), and post-fixed with 4% paraformaldehyde prior to hybridization. Hybridization was done overnight at 37°C. Tissues were washed with the manufacturer’s wash buffer, and then 5XSSCT (standard sodium citrate with 0.1% Tween-20), and the amplification/chain reaction steps were performed following the manufacturer’s protocol. Probe sets were designed and generated by Molecular Instruments and can be ordered directly from their website.

### 2.8. Confocal microscopy

Whole, HCR-processed, adult (0.5–1.5 years) retinas were mounted in glycerol and imaged with a 20X dry lens, 40X water-immersion lens, or 40x oil-immersion lens. 3 μm-step sizes were used for 20X images. Z-series for 40x images were taken with ≤1 μm step size. Whole larval eyes were removed from HCR-processed embryos, and the sclera removed by microdissection. Eyes were mounted in glycerol and imaged with a 20X dry lens using a Nikon-Andor spinning disk confocal microscope and Zyla sCMOS camera. A z-series encompassing the entire globe of the embryo eye was obtained with 3-μm step sizes, using Nikon Elements software. Z-stacks were flattened by max projection, and brightness/contrast adjusted in FIJI (ImageJ). Cross sectional images for adult whole mounted retinas were obtained using the orthogonal view function in FIJI.

Presence/absence of *gngt2a* expression in dorsal regions of larval eyes was determined using whole eye stacks. Brightness/contrast were adjusted to determine presence/absence of signal when signal was dim. Presence within dorsal retina was defined as signal localized in the center of the region dorsal to the center of the lens. A proportion test was used to determine statistical significance.

Hybridization chain reaction fluorescence intensity quantification was performed using FIJI, using the approach of Thiel et al. (2022). For each image the color channels were split, and background measurements were performed on the channel reporting expression of the gene of interest at 3 positions in the z dimension. Whole eyes were traced in the segmentation editor to create an “object” for measurement. The 3D intensity measure plugin was used to obtain “intensity sum,” also known as integrated density, as well as total volume. The single channel reporting expression of the gene of interest was used as the “signal” for 3D analysis. The mean fluorescence intensity of the background was multiplied by the volume of the eye to determine total background. Corrected total fluorescence was calculated by subtracting total background from measured intensity sum (Thiel et al., 2022).

Graphing and statistics were performed in Excel. Sample groups were evaluated for normal distributions using the Shapiro–Wilk test. For comparisons showing normal distributions, *p*-values were calculated using two-tailed Student’s *t*-test, and for comparisons not showing normal distributions, *p*-values were calculated using Mann–Whitney tests. ^***^ denotes *p* < 0.001, ^**^ denotes *p* < 0.01, * denotes *p* < 0.05.

## 3. Results

### 3.1. Transcriptome analysis of LWS1 vs. LWS2 Cones: FACS-bulk-RNA-Seq

**FIGURE 1:**
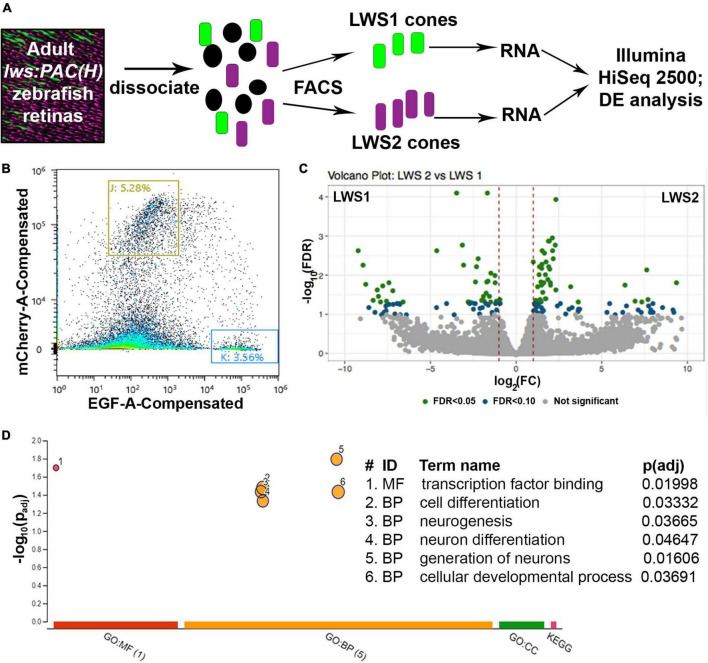
Comparative transcriptome analysis of LWS1 vs. LWS2 cones using FACS followed by bulk RNA-Seq. **(A)** Schematic of dissociation and sequencing workflow. **(B)** Representative (100,000 sorted events) sorting report for an *lws:PAC(H)* sample used in the study; red fluorescence intensity vs. green fluorescence intensity. Gating strategy (boxes labeled J and K) for this sort resulted in the sorting percentages of events indicated. **(C)** Volcano plot depicting transcripts differentially expressed (DE) in LWS1 (left side) vs. LWS2 (right side) cones. Green symbols, transcripts DE at FDR < 0.05; blue symbols, transcripts DE at FDR < 1.0. **(D)** Gene ontology (GO) analysis depicting GO categories overrepresented in the list of DE genes enriched in LWS1 (vs. LWS2) cones. MF, molecular function; BP, biological process.

To address the hypothesis that LWS1 and LWS2 cone subtypes of the zebrafish are distinct in transcriptional characteristics other than opsin expression, we aimed to identify any genes that are DE in these two cone populations of adult zebrafish. LWS1 vs. LWS2 cones of the *lws*:PAC(H) transgenic line were FACS-sorted based upon GFP vs. RFP fluorescence (Sun et al., 2018b), and RNA isolated from the sorted cones was sequenced to discover their respective transcriptomes (Figures 1A, B). The results of the dissociation, sorting, evaluation of purity, and RNA quality were reported earlier (Sun et al., 2018b). Based upon a false discovery rate (FDR) < 0.05, and absolute fold-change (FC) > 2, 95 transcripts were enriched in GFP + (LWS1) cones, representing ∼0.6% of the LWS1 transcriptome (Figure 1C, Table 1, and Dataset 1. Note that Tables 1, 2 and Datasets 1–3 show log_2_FC rather than absolute FC). Using the same cutoff criteria, 186 transcripts were enriched in RFP + (LWS2) cones, representing ∼1.2% of the LWS2 transcriptome (Figure 1C, Table 2, and Dataset 1). These analyses suggest that these cone subtypes are indeed highly similar, although with sufficient transcriptional differences other than opsin expression, to support our original hypothesis. A moderated Log_2_FC approach, to normalize for transcripts expressed at very low levels, returned fewer, but several of the same transcripts (Dataset 2; 19 genes in common with Dataset 1 as LWS1-enriched; 56 genes in common with Dataset 1 as LWS2-enriched). The entire dataset is publicly accessible (GEO accession #GSE232902).^3^

**TABLE 1:** Selected genes differentially expressed (enriched) in LWS1 (GFP +) vs. LWS2 (RFP +) cones.

Gene_name	Gene_description	Log_2_FC	adj. *P*-value
*gngt2a*	Guanine nucleotide binding protein (G protein), gamma transducing activity polypeptide 2a	-1.36672	3.90E-12
*nrip1a*	Nuclear receptor interacting protein 1a	-2.04606	1.69E-05
*per1b*	Period circadian clock 1b	-1.04133	2.08E-05
*cry1bb*	Cryptochrome circadian clock 1bb	-1.92388	3.12E-05
*snap25a*	synaptosomal-associated protein, 25a	-1.40065	0.000127
*neurod1*	Neuronal differentiation 1	-1.3581	0.000382
*taok2a*	TAO kinase 2a	-1.03578	0.000645
*foxg1a*	Forkhead box G1a	-2.25344	0.000732
*ppef2b*	Protein phosphatase with EF-hand domain 2b	-1.82645	0.000732
*hmgb1b*	High mobility group box 1b	-1.2662	0.000749
*znf395a*	Zinc finger protein 395a	-2.99349	0.000798
*per3*	Period circadian clock 3	-1.16867	0.002191
*plxna1a*	Plexin A1a	-1.29707	0.003367
*efna1a*	Ephrin-A1a	-1.36806	0.005018
*neurod4*	Neuronal differentiation 4	-1.90268	0.00563
*acvr1ba*	Activin A receptor, type IBa	-1.67671	0.00563
*thrab*	Thyroid hormone receptor alpha b	-1.17058	0.00563
*pkp1b*	Plakophilin 1b	-1.56174	0.00644
*nlgn4b*	Neuroligin 4b	-2.07195	0.011266
*hmgb3a*	High mobility group box 3a	-1.12235	0.01199
*ntm*	Neurotrimin	-1.72035	0.022466
*rorcb*	RAR-related orphan receptor C b	-1.66891	0.030361
*vax1*	Ventral anterior homeobox 1	-2.44783	0.034141
*opn1lw1*	opsin 1 (cone pigments), long-wave-sensitive 1	-1.72355	0.291626

**TABLE 2:** Selected genes differentially expressed (enriched) in LWS2 (RFP +) vs. LWS1 (GFP +) cones.

Name	Gene description	Log_2_FC	adj. *P*-value
*psmb1*	Proteasome subunit beta 1	2.39001	1.09E–06
*LOC562466*	Cyclic nucleotide-gated cation channel beta-3-like	1.729221	1.81E–05
*calm1b*	Calmodulin 1b	2.011385	2.08E–05
*adgrl3.1*	Adhesion G protein-coupled receptor L3.1	1.603454	0.000213
*gngt2b*	Guanine nucleotide binding protein (G protein), gamma transducing activity polypeptide 2b	1.849842	0.000231
*nptna*	Neuroplastin a	2.224251	0.002037
*nr2f2*	Nuclear receptor subfamily 2, group F, member 2	1.693942	0.00244
*sox4a*	SRY (sex determining region Y)-box 4a	2.357806	0.005018
*nr4a3*	Nuclear receptor subfamily 4, group A, member 3	2.099178	0.01027
*ift27*	Intraflagellar transport 27 homolog (Chlamydomonas)	1.581392	0.011241
*syt4*	Synaptotagmin IV	7.886233	0.02799
*stox2a*	Storkhead box 2a	1.827619	0.028404

Transcripts enriched in LWS1 cones included those with functions in phototransduction [*gngt2a*, encoding one of the γ subunits of transducin; (Lagman et al., 2015)], circadian rhythms (*per1b*, *cry1bb*), cell adhesion (*plxna1a*, *ephrin-A1a*), and transcriptional regulation (*foxg1a*, *hmgb1b*, *rorcb*) (Table 1 and Datasets 1, 2). Notable LWS1-enriched transcripts included two with functions related to TH signaling (*nrip1a*, *thrab*), which is a powerful regulator of *lws1* vs. *lws2* expression (Mackin et al., 2019). A gene ontology (GO) analysis returned one overrepresented molecular function category (transcription factor binding), and several cellular process categories related to differentiation and neurogenesis (Figure 1D). Transcripts enriched in LWS2 cones also included those with functions in phototransduction (*gngt2b*, *cngb3*), cell adhesion (*adgrl3.1*, *nptna*), and transcriptional regulation related to nuclear hormone signaling (*nr2f2*, *nr4a3*), although none with functions in circadian rhythms (Table 2 and Datasets 1, 2). GO analysis of LWS2-enriched transcripts will be discussed below. Therefore, in addition to the divergent λ_max_ of LWS1 vs. LWS2 cones (Chinen et al., 2003), the two populations may have further distinctions in phototransduction kinetics, cell-cell contacts, and transcriptional regulation by nuclear hormone receptors.

Selected transcripts were analyzed from two additional sorting experiments by qPCR. The results from Sort #1 were previously reported, and validated the presence of *opn1lw1*, but absence of *opn1lw2* (along with two *rh2*-type opsin transcripts) in the GFP + (LWS1) cones, and the presence of *opn1lw2*, but absence of *opn1lw1* (along with two *rh2*-type opsin transcripts) in LWS2 cones (Sun et al., 2018b). Sort #2 verified depletion of *gngt2a*, *arr3a*, and *neurod1*, and enrichment of *opn1lw2* and *gngt2b* in LWS2 (RFP +) cones (Supplementary Figure 1). Curiously, *opn1lw1* was not detected as DE by either the RNA-Seq analysis, or by qPCR of Sort #2 (Supplementary Figure 1). Although we only rarely observe co-expression of GFP and RFP reporters in adult *lws*:PAC(H) retinas (Tsujimura et al., 2010; Mitchell et al., 2015; Stenkamp et al., 2021), we tested whether co-expression was more common for the native transcripts, using multiplex fluorescence *in situ* hybridization. These studies confirmed that a small fraction of the adult LWS cones indeed express both *lws* transcripts. Further, we note that the fish used for the RNA-Seq studies were sacrificed in the morning, a time of reduced levels of cone opsin transcript expression (Li et al., 2008), perhaps affecting the likelihood of detecting *lws1* as DE.

Another feature of the DE list that represents a possible limitation of the FACS-RNA-Seq approach is the abundance of DE transcripts encoding components of the proteasome (37 of the 185 transcripts enriched in LWS2 cones; Datasets 1, 2). Correspondingly, GO analysis returned numerous overrepresented categories, including one KEGG category related to proteasomal function (Supplementary Figure 2A) The “RFP” in *lws*:PAC(H) is dsRedExpress (Tsujimura et al., 2010), which has been noted to mis-fold and/or aggregate (Stenkamp et al., 2021), and engage cell stress pathways (Zhou et al., 2011), and so this is a potential explanation for the enriched presence of proteasome components.

We therefore used an additional FACS-RNA-Seq approach and analysis, to validate our findings and identify more transcripts enriched in LWS1 vs. LWS2 cones. Both types of LWS cones were sorted from *thrb2*:tdTomato transgenic retinas, using the strategy reported in Sun et al. (2018b); (Supplementary Figures 2B, C). Subsequent RNA-Seq (GEO upload in progress) and DE analyses using the *thrb2*:tdTomato transcripts vs. the *lws1*:GFP or *lws2*:RFP transcripts returned lists of transcripts DE in LWS1 cones vs. all LWS cones, and in LWS2 cones vs. all LWS cones (Dataset 3 and Supplementary Figure 2D). Not surprisingly, the list of transcripts enriched in LWS2 cones vs. all LWS cones was dominated by components of the proteasome; however, both lists contained transcripts identified in the prior analysis as DE, and also returned some novel findings. The additional dataset is also publicly available (GEO accession # GSE232902) (see text footnote 3).

### 3.2. Transcriptome analysis of LWS1 vs. LWS2 Cones: scRNA-Seq

**FIGURE 2:**
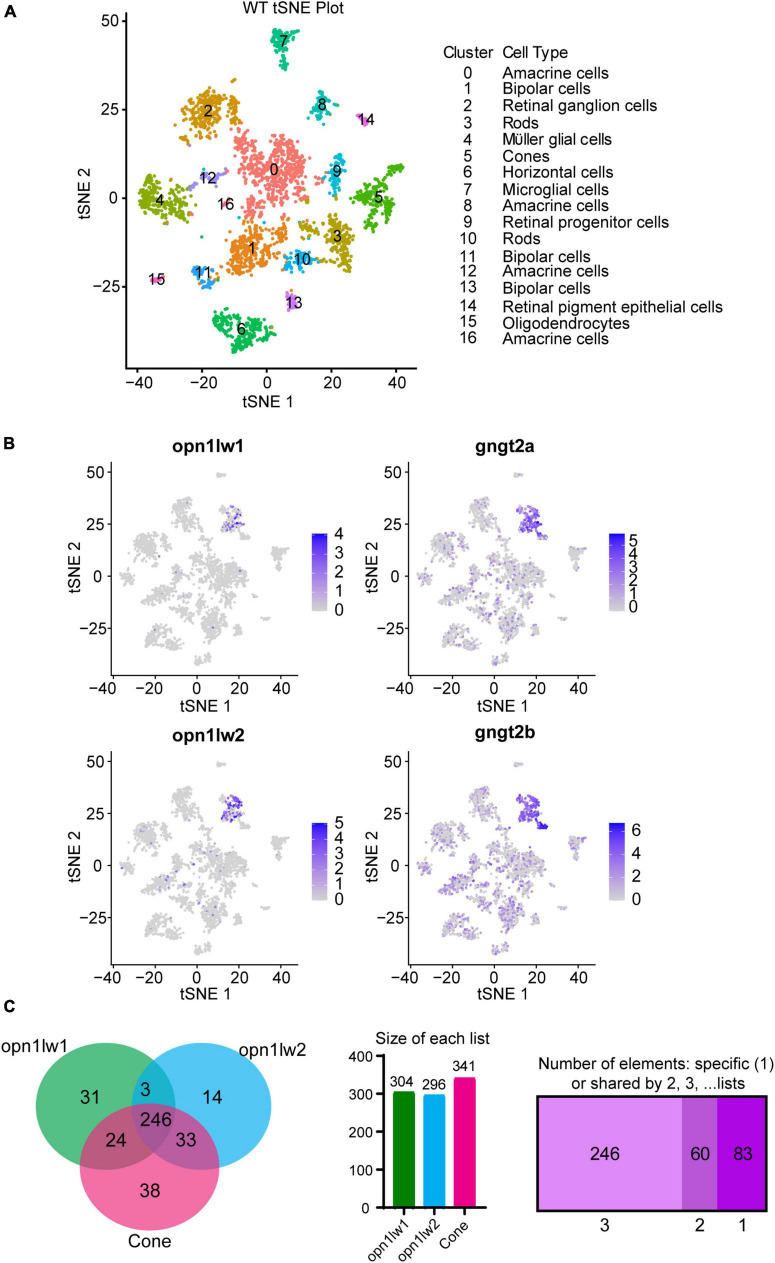
Single cell RNA-Seq and interrogation for transcripts enriched in LWS1 vs. LWS2 cones. **(A)** Visualization of scRNA-Seq output using t-distributed stochastic neighbor embedding (TSNE) plots. Colors of plotted symbols correspond to retinal cell types as predicted by gene expression. **(B)** Expression of *lws1* (*opn1lw1*), *lws2* (*opnlw2*), *gngt2a*, and *gngt2b* predominantly within the cone cluster of the TSNE plot. There is very little coincidence in *lws1* vs. *lws2* expression in individual cones, but greater coincidence for *gngt2a* and *gngt2b*. **(C)** Venn diagram of genes specifically expressed by LWS1 cones, LWS2 cones, and the overall cone population. scRNA-Seq was able to identify transcripts unique to each LWS cone subtype as well as those common to both. Individual lists of transcripts within each space of the Venn diagram are provided as Dataset 4.

As a further means to assess transcriptional distinctions between LWS1 and LWS2 cones, we used scRNA-Seq of WT adult zebrafish retinas. TSNE plotting identified 16 distinct clusters from dissociated retinal cells (Figure 2A), with cone identity assigned to cluster#5, based upon expression of known cone transcripts: opsin markers, *gnat2*, and *pde6c*. Within this cluster, individual cells could be identified based upon expression of *opn1lw1* (94 cells) or *opn1lw2* (199 cells) (Figure 2B). These cells were localized in the TSNE space near each other, and some *lws2* transcript was present in some LWS1 cones, suggesting that cones co-expressing *lws1* and *lws2* were sampled in this study (Figure 2). All transcripts identified within these cones included 573 in the *lws1* + cells (31 unique to *lws1* +), and 886 in the *lws2* + cones (13 unique to *lws2* +) (Dataset 4). The scRNA-Seq dataset is publicly accessible (GEO accession #GSE234661).^4^

A DE analysis of *lws1* + cells vs. *lws1*- cells of the scRNA-Seq results provided another means of identifying transcripts enriched in LWS1 cones vs. other retinal cell types (Dataset 4). Transcripts enriched in LWS1 cones included many identified by the bulk RNA-Seq approach, such as *gngt2a*, *arr3a*, and *ablim3*, as well as those not in the bulk RNA-Seq DE lists, including *aanat21* and *si:busm1-57f23.11* (Figure 2C and Dataset 4). Transcripts enriched in LWS2 cones vs. other retinal cell types included *gngt2b*, *thrb1*, and *six7*. The most consistently identified DE transcripts in LWS1 vs. LWS2 cones, using both approaches, were the two paralogs encoding γ subunits of cone transducin, with *gngt2a* enriched in LWS1 cones, and *gngt2b* enriched in LWS2 cones. Known expression patterns of these paralogs appeared to support some degree of cone subtype specificity, with *gngt2a* found in ventral/peripheral retina, and *gngt2b* found in dorsal/central larval retina (Lagman et al., 2015), patterns shown to reflect those of *lws1* and *lws2*, respectively (Takechi and Kawamura, 2005; Tsujimura et al., 2010; Ogawa and Corbo, 2021). The mapping of *gngt2a* and *gngt2b* paralogs on the TSNE graph also suggested coordinated co-expression of these transcripts within specific LWS cone subtypes, although the *gngt2*s were also more broadly associated with other retinal cell type clusters (Figure 2B).

We wished to further evaluate potential DE transcripts through curating a “short list” to prioritize for additional study (Table 3). Transcripts were prioritized based upon (1) appearing in more than one DE list; (2) evidence in the literature (or in the ZFIN database) of expression patterns consistent with DE in LWS1 vs. LWS2 cones; (3) levels of expression in the bulk RNA-Seq dataset suggesting the transcript would be detectable by *in situ* hybridization; (4) potential cone-specific functional relevance (phototransduction, circadian rhythm, cell adhesion); and (5) potential relevance in regulation of *lws1* vs. *lws2* expression (nuclear hormone signaling) (Mitchell et al., 2015; Mackin et al., 2019). We noted that *si:busm1-57f23.1* within our short list, was also detected as significantly downregulated in a zebrafish *thrb* mutant (Volkov et al., 2020). We reasoned that other DE transcripts identified in *thrb-/-* vs. WT in this previous study may also be DE in LWS1 vs. LWS2 cones, and so added these to the short list.

**TABLE 3:** Curated “short list” of transcripts DE in LWS1 (vs. LWS2) or LWS2 (vs. LWS1) prioritized for further study.

Name	Subtype enriched	Predicted/Known function	Data predicting enrichment
*gngt2a*	LWS1	Phototransduction	Dataset 1, 4
*gngt2b*	LWS2	Phototransduction	Dataset 1, 2, 4
*nrip1a*	LWS1	Nuclear receptor interacting	Dataset 1
*nr2f2*	LWS2	Nuclear receptor	Dataset 1, 2
*vax1*	LWS1	Transcription factor	Dataset 1
*vax2*	LWS1	Transcription factor	Dataset 1
*si:busm1*	LWS1	Endopeptidase inhibitor	Dataset 4
*cry3a*	LWS1	Circadian rhythm	Dataset 4
*six7*	LWS1	Transcription factor	Dataset 1, 4
*irbp*	LWS2	Retinoid binding	Dataset 1
*rbp41*	LWS2	Retinoid binding	Dataset 1, 4
*sox4a*	LWS2	Transcription factor	Dataset 1
*dio3b*	LWS1	TH inactivator	Dataset 1, 2, 4

### 3.3. Expression analysis of LWS1-enriched and LWS2-enriched transcripts in adult retinas

We selected eight of these transcripts (Table 2; LWS1-enriched: *gngt2a*, *nrip1a*, *vax1*, *vax2*, *si:busm1*, *cry3a*. LWS2-enriched: *gngt2b*, *nr2f2*) to test two hypotheses: (1) LWS1 and LWS2 cones are functionally distinct (already supported by the outcomes of the RNA-Seq analyses); and (2) multiple genes are coordinately regulated in LWS cones by thyroid hormone. We also wished to use these transcripts to evaluate heterogeneity within the LWS cone subtypes, as such heterogeneity was noted by Aramaki et al. (2022) for M opsin dominant vs. S opsin dominant cone types in mouse. Our initial approach was to focus upon the first hypothesis by evaluating expression patterns of these transcripts in whole adult wild-type retinas using multiplex *in situs*.

#### 3.3.1. gngt2a and gngt2b

**FIGURE 3:**
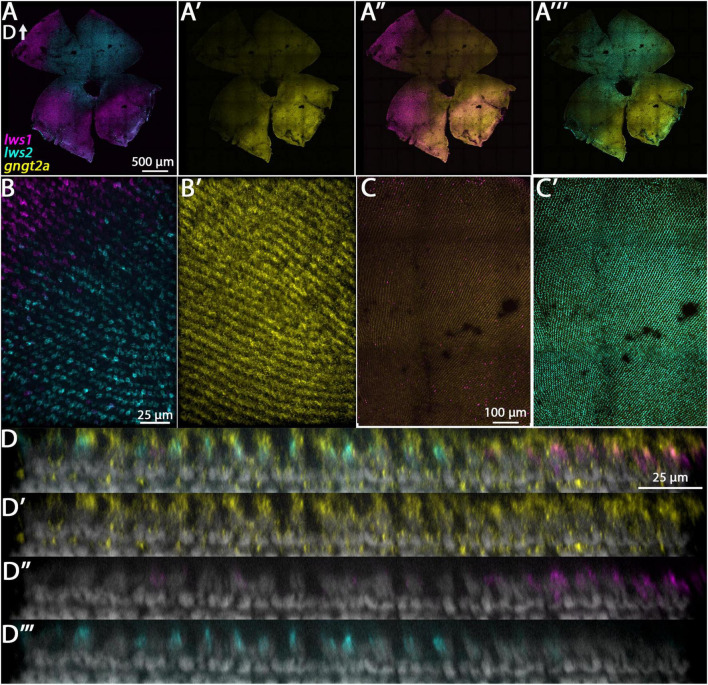
Expression of *gngt2a* in adult wildtype zebrafish retina using multiplex fluorescence *in situ* hybridization chain reaction (HCR). **(A)** Expression of *lws1* (magenta) and *lws2* (cyan) in a representative whole retina (D, dorsal). **(A’)** Expression of *gngt2a* (yellow) in the same preparation, showing that signal intensity appears greatest in the *lws1*-expressing domain, but signal is not confined to this domain. **(A”)** *gngt2a* and *lws1*. **(A”’)** *gngt2a* and *lws2*. **(B)** 40x image of *lws1* and *lws2* expression in a region of transition from *lws1* to *lws2*. **(B’)** 40x image of *gngt2a* expression in the same region, indicating *gngt2a* is not restricted to *lws1*-expressing cones, nor to LWS cones in general. **(C)** Selected enlarged region of **(A”)** showing *gngt2a* and *lws1*. **(D)** Same region showing *gngt2a* and *lws2*. **(D–D”’)** Resliced orthogonal projections of **(B)**. **(D)** All imaging channels merged. **(D’)** DAPI and *gngt2a.* **(D”)** DAPI and *lws1*. **(D”’)** DAPI and *lws2. gngt2a* is co-expressed by *lws1* + and *lws2* + cones, as well as in non-LWS cones. Sample size = 2.

**FIGURE 4:**
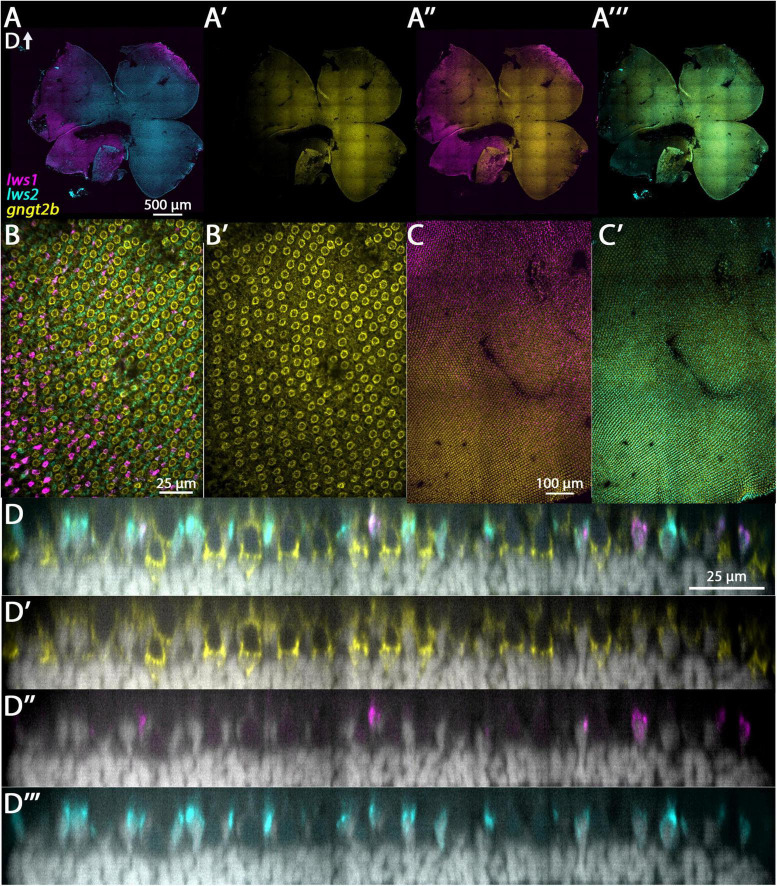
Expression of *gngt2b* in adult wildtype zebrafish retina using HCR. **(A)** Expression of *lws1* (magenta) and *lws2* (cyan) in a representative whole retina. **(A’)** Expression of *gngt2b* (yellow) in the same preparation, showing that signal intensity appears greatest in the *lws2*-expressing domain. **(A”)** *gngt2b* and *lws1*. **(A”’)** *gngt2b* and *lws2*. **(B)** 40x image of *lws1*, *lws2*, and *gngt2b* expression in a region of transition from *lws1* to *lws2*, indicating *gngt2b* and *lws2* co-expression, and *gngt2b* expression in other populations of photoreceptors. **(B’)** 40x image of *gngt2b* expression, highlighting distinct subcellular expression domains of *gngt2b*. **(C)** Selected enlarged region (transition zone) of **(A”)** - *gngt2b* and *lws1*. **(D)** Same region showing *gngt2b* and *lws2*. **(D–D”’)** Resliced orthogonal projections of panel **(B)**. **(D)** All imaging channels merged. **(D’)** DAPI and *gngt2b.* **(D”)** DAPI and *lws1*. **(D”’)** DAPI and *lws2.* D, dorsal. Samples size = 2.

The genes *gngt2a* and *gngt2b* are paralogous and encode gamma subunits of the heterotrimeric g-protein transducin, an essential part of the phototransduction cascade (Lagman et al., 2015). Previous studies have shown that the expression domains of *gngt2a* and *gngt2b* correspond with the expression domains of *lws1* and *lws2*, respectively (Takechi and Kawamura, 2005; Ogawa and Corbo, 2021). Consistent with these studies, our RNA-Seq data show that *gngt2a* is enriched in LWS1 cones and *gngt2b* is enriched in LWS2 cones (Tables 1, 2 and Datasets 1, 2, 4). While the majority of *gngt2a* and *gngt2b* expressing cones are found within zones of *lws1* and *lws2* expression, respectively, multiplex fluorescence in situs of whole mounted adult retinas show expression of *gngt2a* and (to a lesser extent) *gngt2b* beyond their respective corresponding *lws* domains (Figures 3A’, 4A’). The expression domain of *gngt2a* was particularly widespread, including nearly the entirety of the retina, although the fluorescence signal appeared stronger in ventral retina (Figure 3A’). The images also show expression of both *gngt2a* and *gngt2b* in non-LWS cones (Figures 3D, 4D). Interestingly, the morphology and position of some of the *gngt2b*-expressing non-LWS cones suggests they may be UV (*sws1*-expressing) cones, as these cells are short, single cones (Figure 4D).

#### 3.3.2. nrip1a and nr2f2

**FIGURE 5:**
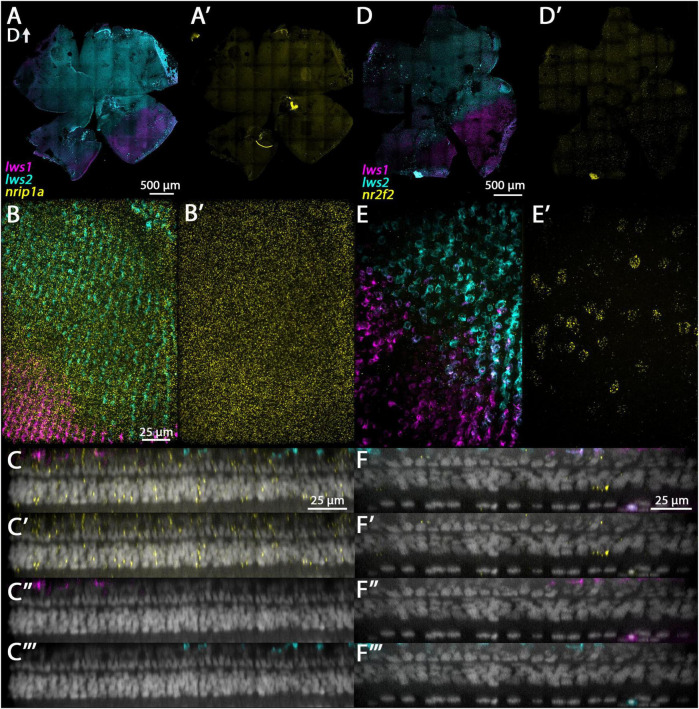
Expression of *nrip1a* and *nr2f2* in adult zebrafish retina. **(A–C”’)** *nrip1a*. **(D–F”’)** *nr2f2*. **(A)** Expression of *lws1* (magenta) and *lws2* (cyan) in a representative whole retina. **(A’)** *nrip1a* expression (yellow) in the same preparation, showing pan-retinal expression. **(B)** 40x image of *lws1*, *lws2*, and *nrip1a* expression in a region of *lws1* to *lws2* transition. **(B’)** *nrip1a* alone, in same region. C-C”’) Resliced orthogonal projection of panel **(B)**. **(C)** All imaging channels merged. **(C’)** DAPI and *nrip1a*. **(C”)** DAPI and *lws1.* **(C”’)** DAPI and *lws2.* **(D)** Expression of *lws1* (magenta) and *lws2* (cyan) in a representative whole retina. D’) *nr2f2* expression (yellow), showing slight bias in signal intensity toward dorsal retina. **(E)** 40x image of *lws1*, *lws2*, and *nr2f2* expression in a region of *lws1* to *lws2* transition, at the level of cone inner segments. Scale same as in panel **(B)**. **(E’)** *nr2f2* alone, in the same region, at the level of the deep INL. **(F–F”’)** Resliced orthogonal projection of panels **(E,F)** All imaging channels merged. **(F’)** DAPI and *nr2f2*. **(F”)** DAPI and *lws1.* **(F”’)** DAPI and *lws2.* D, dorsal. Sample size = 2.

The protein encoded by *nrip1a* is predicted to interact with nuclear hormone receptors (O’Leary et al., 2016; Grasedieck et al., 2022), and is expressed in the anterior nervous system of zebrafish embryos (Thisse and Thisse, 2008). The bulk RNA-Seq of sorted LWS1 vs. LWS2 cones, and the analysis of the scRNA-Seq output, indicated that *nrip1a* transcript is enriched in LWS1 cones (Table 1 and Datasets 1, 4). Multiplex in situs of adult whole retinas instead show widespread expression of *nrip1a* across the retina, without a ventrally-biased pattern, as would be predicted from the bulk-RNA-Seq (Figure 5A’). The *nrip1a* transcript is indeed present in LWS cones of both types, and also in non-LWS cones (Figure 5C). *Nrip1a* also appears to be expressed in cells of other retinal layers (Figure 5C). *Nr2f2* encodes a nuclear hormone receptor with several known patterning roles within the nervous system and other organs (Barske et al., 2018), and is expressed within the photoreceptor layer of zebrafish embryos (Thisse and Thisse, 2008). Our bulk RNA-Seq and scRNA-Seq results indicate that *nr2f2* transcript is enriched in LWS2 cones (Table 2 and Datasets 1, 2). Multiplex in situs of adult whole retina show a very slight bias in the *nr2f2* expression domain toward the dorsal half of the retina (Figure 5A), similar to the *lws2* expression domain but with a less abrupt transition (Figure 5D’). Further, our results show *nr2f2* is expressed in LWS cones (both LWS1 and LWS2) as well as in some cells of the INL having positions consistent with the identity of amacrine cells (Figure 5F).

#### 3.3.3. vax1 and vax2

**FIGURE 6:**
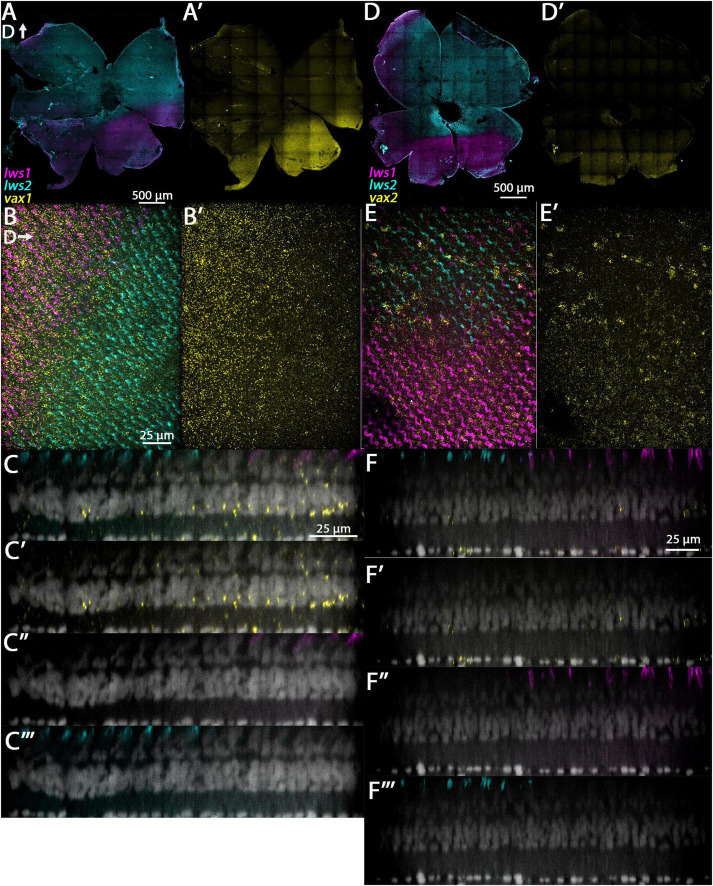
Expression of *vax1* and *vax2* in adult zebrafish retina. **(A–C”’)** *vax1*. **(D–F”’)** *vax2*. **(A)** Expression of *lws1* (magenta) and *lws2* (cyan) in a representative whole retina. **(A’)** *vax1* expression (yellow) in the same preparation, showing expression restricted to the *lws1*-expressing domain. **(B)** 40x image of *lws1*, *lws2*, and *vax1* expression, in a region of *lws1* to *lws2* transition; orientation of panels **(B,B’)** are such that dorsal is to the right. **(B’)** 40x image of *vax1* alone, showing transition to *vax1* domain is less abrupt than the transition to the *lws1* domain. **(C–C”’)** Resliced orthogonal projections of panel **(B)**. **(C)** All imaging channels merged. **(C’)** DAPI and *vax1*. **(C”)** DAPI and *lws1.* **(C”’)** DAPI and *lws2.* **(D)** Expression of *lws1* (magenta) and *lws2* (cyan) in a representative whole retina. **(D’)** *vax2* expression (yellow) in the same preparation, showing expression restricted to the *lws1*-expressing domain. **(E)** 40x image of *lws1*, *lws2*, and *vax2* expression, in a region of *lws1* to *lws2* transition. **(E’)** 40x image of *vax2* alone in the same region, showing transition to *vax1* domain is less abrupt than the transition to the *lws1* domain. **(F–F”’)** Resliced orthogonal projections of panel **(E)**. **(F)** All imaging channels merged. **(F’)** DAPI and *nr2f2*. **(F”)** DAPI and *lws1.* **(F”’)** DAPI and *lws2.* D, dorsal. Sample size = 2.

These genes encode transcription factors needed for optic cup morphogenesis and closure of the choroid fissure (Take-uchi et al., 2003) and are expressed in ventral regions of the embryonic zebrafish retina (Holly et al., 2014; Richardson et al., 2019). Our bulk and scRNA-Seq data indicate that *vax1* is enriched in LWS1 cones (Table 1 and Dataset 1). Multiplex *in situ* of adult zebrafish retinas verify that its expression domain is restricted to the ventral portion of the retina (Figure 6A’). Interestingly, the *vax1* and *lws1* expression domains were very similar (Figures 6A’, D’). Resliced orthogonal projections show *vax1* expression in cells of the photoreceptor layer, including LWS1 cones, INL, and some cells of the GCL, with strongest signal localized to the INL (Figure 6C). *Vax2* transcripts are also present in LWS1 cones, though not significantly enriched, based upon the output of our RNA-Seq analyses (Table 1 and Dataset 1) [see also (Ogawa and Corbo, 2021)]. Whole mounted retinas processed for multiplex in situs show its expression is limited to a ventral portion of the retina (Figure 6D’). Orthogonal views reveal expression of *vax2* in cells of the photoreceptor layer, including LWS1 cones, the INL, and the GCL, with most of the expression in the INL and GCL (Figure 6F).

#### 3.3.4. si:busm1-57f23.1

**FIGURE 7:**
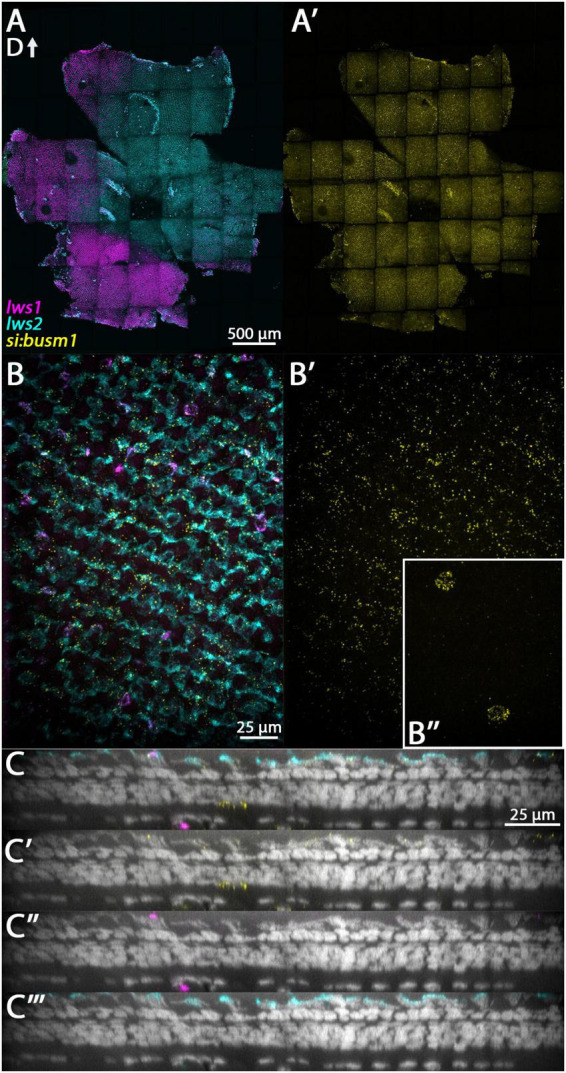
Expression of *si:busm1* in adult zebrafish retina. **(A)** Expression of *lws1* (magenta) and *lws2* (cyan) in a representative whole retina. **(A’)** *si:busm1* expression (yellow) in the same preparation showing pan-retinal expression. **(B)** 40x image of *lws1*, *lws2*, and *si:busm1* expression in a region of *lws1* to *lws2* transition. **(B’)** 40x image of *si:busm1* alone. Inset continues the field of view but at the level of the boundary between the inner nuclear layer and inner plexiform layer. **(C–C”’)** Resliced orthogonal projections of panel **(B)**. **(C)** All imaging channels merged. **(C’)** DAPI and *si:busm1*. **(C”)** DAPI and *lws1.* **(C”’)** DAPI and *lws2.* D, dorsal. Sample size = 2.

The gene *si:busm1-57f23.1* encodes a protein that is predicted to be a secreted endopeptidase inhibitor (O’Leary et al., 2016), and in zebrafish embryos transcript is expressed in the photoreceptor layer of the retina (Hartmeyer et al., 2004). Transcripts are predicted to be highly enriched in LWS cones of adult zebrafish, since a *thrb* mutant lacking LWS cones displays very low levels of expression in comparison with wildtype (Volkov et al., 2020). Our scRNA-Seq results expand on this information, suggesting that this gene is more highly expressed in LWS1 cones than in LWS2 cones (Dataset 4). While multiplex *in situ* images do not show an obvious bias in expression domain toward the LWS1 domain (Figure 7A’), they confirm the presence of *si:busm1-57f23.1* transcript in cones (LWS cones and potentially some non-LWS cones; Figure 7C). This gene also appears to be sporadically expressed by some cells of the INL, possibly amacrine cells (Figure 7C).

#### 3.3.5. cry3a

*Cry3a* encodes a cryptochrome circadian regulator, is expressed within the embryonic zebrafish retina (Tsai et al., 2020), and is indicated by scRNA-Seq dataset to be enriched within LWS1 cones vs. LWS2 cones (Dataset 4). Images of adult retina multiplex in situs show this gene is diffusely expressed in the adult zebrafish retina and is present in all retinal layers (Supplementary Figure 3).

In summary, multiplex *in situ* hybridization supported the findings from the bulk and scRNA-Seq indicating that the eight transcripts evaluated were indeed expressed in LWS cones. Further, the in situs supported that *gngt2a*, *vax1*, and *vax2*, but not *nrip1a*, *si:busm1-57f23.1*, and *cry3a* are enriched in LWS1 vs. LWS2 cones, and that *gngt2b* and *nr2f2* are enriched in LWS2 vs. LWS1 cones. Thus, we find further support for the hypothesis that LWS1 cones are transcriptionally distinct from LWS2 cones. In addition, we also observe considerable heterogeneity within these two populations in expression of the predicted enriched transcripts.

### 3.4. Analysis of TH-mediated regulation of LWS1-enriched and LWS2-enriched transcripts

We next used a TH treatment protocol demonstrated to increase *lws1* at the expense of *lws2*, in individual cones (Mackin et al., 2019), to evaluate the response of the eight transcripts to TH, thereby testing our second hypothesis. This protocol involved treatment of zebrafish embryos at 48 hpf with 100 nM T3, or the DMSO vehicle, and collecting whole larvae at 96 hpf for measurement of relative abundance of transcript (qPCR) and changes in expression pattern (multiplex fluorescence *in situ*) as the experimental endpoints.

#### 3.4 1. gngt2a and gngt2b

**FIGURE 8:**
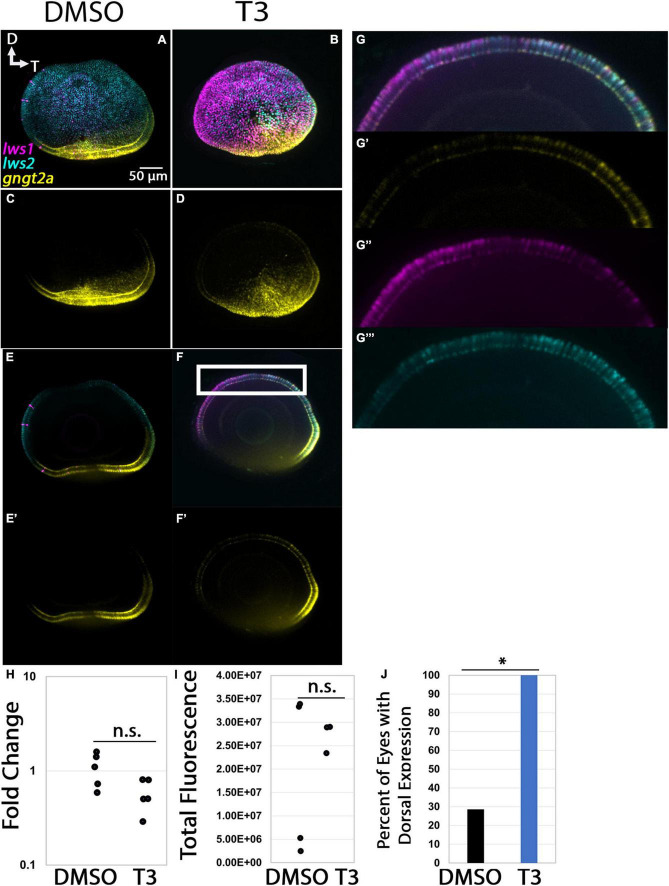
Expression of *gngt2a* in control (DMSO) and TH-treated (T3) larval zebrafish. **(A–D)** Projections of representative whole, imaged eyes. Note reduced expression domain of *lws2* (cyan), and expanded expression domains of *lws1* (magenta) and *gngt2a* (yellow) in T3-treated **(B,D)** vs. controls **(A,C)**; expanded domains do not appear to align, however, **(E–F’)** Single z slices obtained from the same preparations. **(G–G”’)** Enlarged images of region within box in pane **(F)**. **(G)** All imaging channels merged. **(G’)** *gngt2a*. **(G”)** *lws1.* **(G”’)** *lws2.* **(H)** qPCR quantification of *gngt2a* transcript abundance in pooled samples of whole larvae, *n* = 5 biological replicates per condition, *p* = 0.0967. **(I)** 3D fluorescence intensity quantification, *n* = 3 embryos per condition. **(J)** percent of eyes with expression of *gngt2a* in dorsal retina, *n* = 7 (DMSO), 5 (T3), *p* = 0.013. (proportion test). D, dorsal; T, temporal. *, *p* < 0.05; n.s., not significant.

The pattern of expression of *gngt2a* in control, 96 hpf whole eyes appeared largely localized to the photoreceptor layer within ventral retina [Figure 8 (Lagman et al., 2015)], distinct from the results from adult retina (Figure 3). Multiplex *in situ* hybridization using probe sets targeting *gngt2a*, *lws1*, and *lws2*, revealed that many cells in this layer and region expressed *gngt2a*, including those that were *lws1* + and those that were *lws2* + (Figures 8A, C, E). Therefore, although *gngt2a* was consistently detected as enriched in adult *lws1* + vs. *lws2* + cones, the patterns of *gngt2a* and *lws1* at larval stages did not precisely align (Figure 8A). Larval eyes that had been treated with 100 nM T3 showed dorsally expanded domains of *gngt2a* expression, along with dorsally expanded domains of *lws1* and restricted domains of *lws2* (Mackin et al., 2019; Figures 8B, D, F). qPCR, however, showed no difference between treatment groups for relative abundance of *gngt2a* (Figure 8H, *p* = 0.0967). Further, quantitative fluorescence analysis showed no difference between treatment groups (Figure 8I, *p* = 0.454). Interestingly, however, the presence of *gngt2a* expression in the dorsal portion of the eye was significantly more likely to be found in T3 treated eyes (Figure 8J, *p* = 0.013). Therefore, it is likely that the expansion of the expression domain, despite being consistent and obvious, did not alter the average amount of *gngt2a* transcript in the whole eye. In total, *lws1* and LWS1 cone-enriched transcript *gngt2a* both appear to be upregulated, but to different degrees and in not exactly the same domains. This suggests that *lws1* and *gngt2a* are regulated in some way by TH but not in a precisely coordinated manner.

**FIGURE 9:**
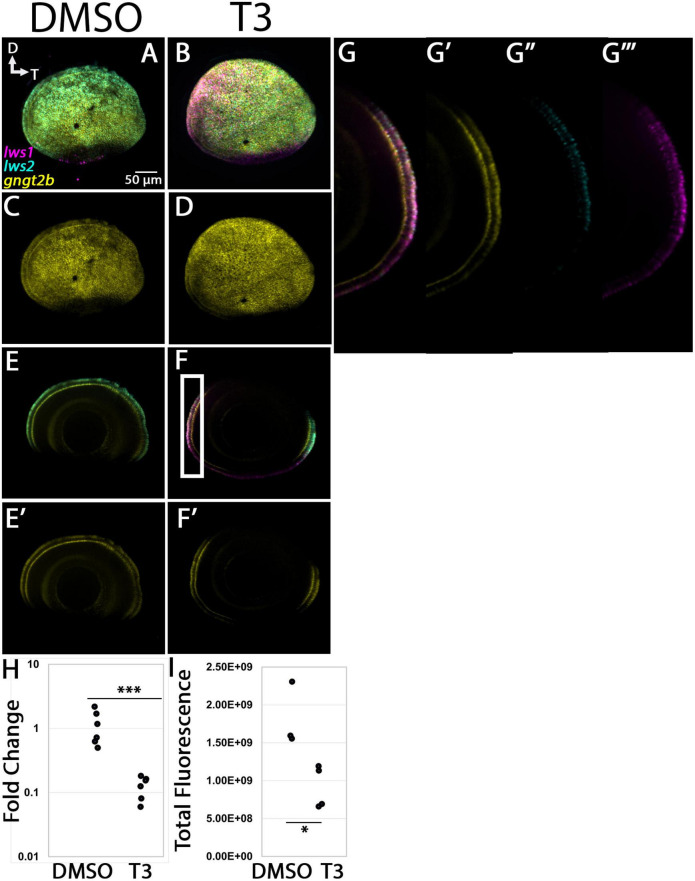
Expression of *gngt2b* in control (DMSO) and TH-treated (T3) larval zebrafish. **(A–D)** Projections of representative whole, imaged eyes. Note reduced expression domain of *lws2* (cyan), and expanded expression domains of *lws1* (magenta) in T3-treated **(B,D)** vs. controls **(A,C)**; the *gngt2b* (yellow) expression domain did not appear to change. **(E–F’)** Single z slices obtained from the same preparations. **(G–G”’)** Enlarged images of region within box in panel **(F)**. **(G)** All imaging channels merged. **(G’)** *gngt2b* **(G”)** *lws2* **(G”’)** *lws1* **(H)** qPCR quantification of *gngt2b* transcript abundance in pooled samples of whole larvae, *n* = 6 biological replicates per condition, *p* = 3.159E-05. **(I)** 3D fluorescence intensity quantification, *n* = 3 embryos (DMSO), 4 (T3). D, dorsal; T, temporal. ^***^, *p* < 0.001; *, *p* < 0.05.

The expression domain of *gngt2b* in 96 hpf whole eyes also appeared localized to photoreceptors, but more widespread than that of *gngt2a*, and excluded from ventral retina [Figures 9A, C (Lagman et al., 2015)], similar to what we observed in adult retina (Figure 4). Many cones co-expressed *lws2* and *gngt2b*, although there were also many *gngt2b* + cells that were not *lws2* + (Figure 9E). The *gngt2b* domain was slightly reduced as a proportion of the eye in comparison with the *lws2* domain, but shared general pattern characteristics. These findings are consistent with the DE analyses of adult LWS1 vs. LWS2 cones. Eyes of larvae treated with T3, somewhat surprisingly, showed no reduction in size of the *gngt2b* expression domain (Figures 9D–G). The fluorescence intensity, however, significantly decreased in the T3 condition, as did transcript abundance reported by qPCR (Figures 9H, I, *p* = 3.159E-05, 0.0191). The size of the *lws2* domain was reduced, and that of the *lws1* domain was enlarged, as expected [Figure 9B; (Mackin et al., 2019)], providing an internal control that the treatment was effective. Whole larval tissues analyzed by qPCR, and whole mounted eyes analyzed by quantitative fluorescence showed increased *lws1* due to T3 treatment (Figures 9H, I). Collectively, these findings support the hypothesis that exogenous TH controls *lws1*, *lws2*, *gngt2a*, and *gngt2b* expression, but not in a topographically, and/or temporally coordinated manner.

#### 3.4 2. nrip1a and nr2f2

**FIGURE 10:**
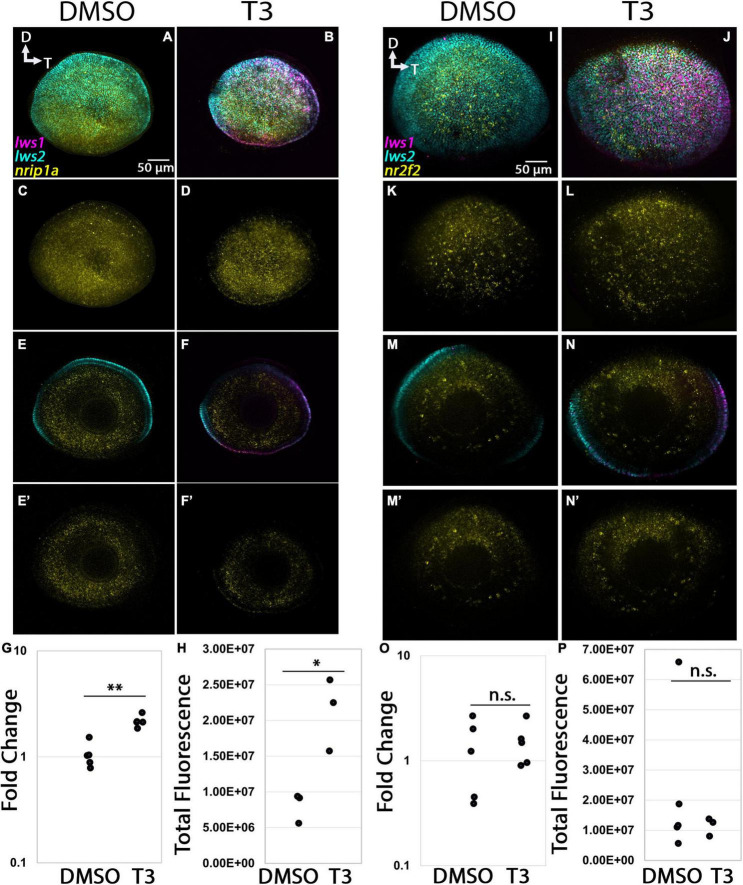
Expression of *nr2f2* and *nrip1a* in larval zebrafish. **(A–H)** *nrip1a*. **(I–P)** *nr2f2*. **(A–D,I–L)** Projections of representative whole, imaged eyes. **(E–F’,M–N’)** Single z slices obtained from the same preparations. Note reduced expression domain of *lws2* (cyan), and expanded expression domains of *lws1* (magenta) in T3-treated **(B,D,J,L)** vs. controls **(A,C,I,K)**; the *nrip1a* (yellow, **A–D**) expression domain did not appear to change; the *nr2f2* (yellow, **I–L**) expression domain did not appear to change, however, there appears to be slightly greater expression in the dorsal portion of the retina **(E,F)** All imaging channels merged. **(E’,F’)** *nrip1a* **(M,N)** All imaging channels merged. **(M’,N’)** *nr2f2* **(G)** qPCR quantification of *nrip1a* transcript abundance in pooled samples of whole larvae, *n* = 5 biological replicates per condition, *p* = 0.476. **(H)** 3D fluorescence intensity quantification for *nrip1a*, *n* = 5 embryos (DMSO), 3 (T3), sample size too small for Mann–Whitney test. **(O)** qPCR quantification of *nr2f2* transcript abundance in pooled samples of whole larvae, *n* = 5 biological replicates per condition, *p* = 0.00152 **(P)** 3D fluorescence intensity quantification of *nr2f2*, *n* = 3 embryos per condition, *p* = 0.014 (*t*-test). D, dorsal, T, temporal. *, *p* < 0.05; **, *p* < 0.01; n.s., not significant.

The *nrip1a* transcript was predicted to be enriched in LWS1 cones, encodes a protein that interacts with nuclear hormone receptors (O’Leary et al., 2016), but adult whole retinas did not display an obvious bias in expression domain (Figure 5). This transcript was abundantly expressed in the retina of 96 hpf larvae, and many cells were co-labeled for *lws1* and *lws2* (Figures 5A–F). T3 treatment did not appear to alter the expression domain of *nrip1a*, although the domains of *lws1* increased, and *lws2* decreased, as expected (Figures 10C, D). Abundance of transcript, however, increased significantly in response to T3 treatment (Figure 10G, *p* = 0.00152). Further, quantitative fluorescence analysis showed increased fluorescence in the T3 treated group, indicating that transcript abundance of *nrip1a* increased upon T3 treatment without altering its expression domain (Figure 10H, *p* = 0.014). The *nr2f2* transcript was predicted to be enriched in LWS2 cones and is a predicted nuclear hormone receptor. This transcript was abundantly expressed in multiple layers of adult retina and displayed a slight bias in expression signal in dorsal retina (Figure 5). At 96 hpf, expression of this transcript also appeared to be higher in the dorsal portion of the retina, which is consistent with the RNA-Seq and adult *in situ* results (Figures 10D, K, M). T3 treatment did not appear to alter the expression domain of this gene (Figures 10J, L, N), and both qPCR and quantitative fluorescence analysis results showed no significant difference between treatment groups (Figures 10O, P, *p* = 0.476, sample size too small for Mann–Whitney test).

#### 3.4 3. vax1 and vax2

**FIGURE 11:**
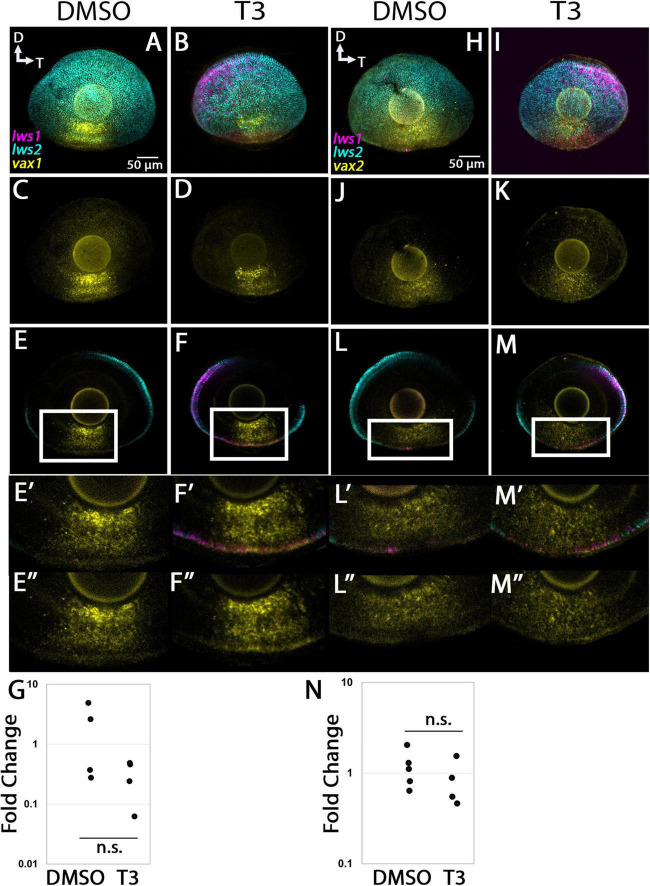
Expression of *vax1* and *vax2* in larval zebrafish. **(A–G)** *vax1*. **(H–N)** *nrip1a*. **(A–D,H–K**) Projections of representative whole, imaged eyes. **(E–F”,L–M”)** Single z slices obtained from the same preparations. Note reduced expression domain of *lws2* (cyan), and expanded expression domains of *lws1* (magenta) in T3-treated **(B,D,I,K)** vs. controls **(A,C,H,J)**; the *vax1* (yellow, **A–D**) and *vax2* (yellow, **H–K**) expression domain did not appear to change. **(E’–F”)** Enlarged images of regions within box of panels **(E,F**, respectively). **(E’,F’)** All imaging channels merged. **(E”,F”)** *vax1* **(L’–M”)** Enlarged images of regions within box of panels **(L,M**, respectively). **(L’,M’)** All imaging channels merged. **(L”,M”)** *vax2* **(G)** qPCR quantification of *vax1* transcript abundance in pooled samples of whole larvae, *n* = 4 biological replicates per condition, *p* = 0.123. **(N)** qPCR quantification of *vax2* transcript abundance in pooled samples of whole larvae, *n* = 5 biological replicates per condition, *p* = 0.312. n.s., not significant.

The *vax1* transcript was predicted to be enriched in LWS1 cones, encodes a transcription factor important in patterning the nervous system, and was expressed in the ventral region of adult retina in multiple retinal layers, with some cells coexpressing *lws1* (Figure 6). T3 treatment did not appear to alter the expression domain of this gene (Figures 11A–F), and qPCR results also showed no significant difference between treatment groups (Figure 11G, *p* = 0.123). The *vax2* transcript was predicted to be enriched in LWS1 cones, encodes a transcription factor important in patterning the nervous system, and was expressed in the ventral region of adult retina in multiple retinal layers, with some cells coexpressing *lws1* (Figure 6). T3 treatment did not appear to alter the expression domain of this gene (Figures 11H–M), and qPCR results also showed no significant difference between treatment groups (Figure 11N, *p* = 0.312).

#### 3.4 4. si:busm1-57f23.1

**FIGURE 12:**
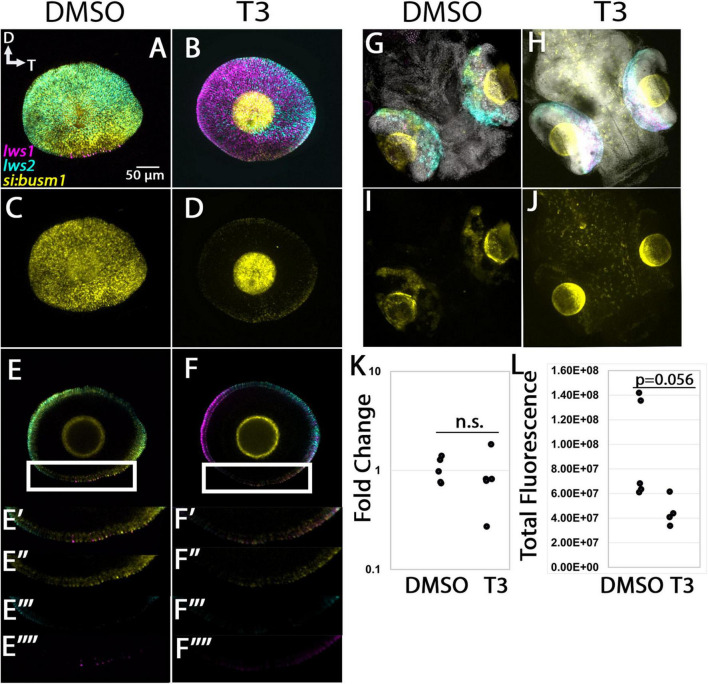
Expression of *si:busm1* in larval zebrafish. **(A–D)** Projections of representative whole, imaged eyes. **(E–F”’)** Single z slices obtained from the same preparations. Note reduced expression domain of *lws2* (cyan), and expanded expression domains of *lws1* (magenta) in T3-treated **(B,D)** vs. controls **(A,C)**; the *si:busm1* (yellow) expression domain appears greatly reduced in the treated condition **(D)**. **(E’–E”’)** Enlarged images of region within box in panel **(E)**. **(E’)** All imaging channels merged. **(E”)** *si:busm1* **(E”’)** *lws2* **(E””)** *lws1* **(F’–F””)** Enlarged images of region within box in panel **(F)**. **(F’)** All imaging channels merged. **(F”)** *si:busm1* **(F”’)** *lws2* **(F””)** *lws1* **(G–J)** Projections of whole embryo heads. **(K)** qPCR quantification of *gngt2a* transcript abundance in pooled samples of whole larvae, *n* = 5 biological replicates, *p* = 0.244. **(L)** 3D fluorescence intensity quantification, *n* = 5 (DMSO), 4 (T3), *p* = 0.056 (*t*-test). D, dorsal, T, temporal. n.s., not significant.

The *si:busm1-57f23.1* transcript was identified as enriched in LWS1 cones by the scRNA-Seq, and is DE (reduced in expression) in *thrb* mutants vs. WT (Volkov et al., 2020). This transcript encodes a predicted extracellular protein with cysteine protease inhibitor activity, and adult retinas indeed show expression in LWS and some non-LWS cones (Figure 7). Expression of *si:busm1-57f23.1* in 96 hpf larvae was localized to the photoreceptor layer, and many cells were co-labeled for *lws1* or *lws2* (Figures 12A–F). Eyes of larvae treated with T3 showed marked reduction in the expression domain of *si:busm1-57f23.1*, as well as reduction in the *lws2* domain and expansion of the *lws1* domain (Figures 12B, D). Whole larval tissues analyzed by qPCR showed no statistically significant change in *si:busm1-57f23.1* expression due to T3 treatment (Figure 12K, *p* = 0.244). We hypothesized that expression in non-retinal tissues may make the apparent downregulation seen in the in situs difficult to detect by qPCR in whole larvae. To check this, we performed whole larval HCR and imaged the entire larval head, including the brain and both eyes. We found that *si:busm1-57f23.1* is, indeed, expressed in the brain and spinal cord of 4 day old zebrafish (Figures 12G–J). We then performed quantitative fluorescence analysis on the eyes alone and found that fluorescence trended down in the eyes of T3 treated embryos (Figure 12L, *p* = 0.0556).

#### 3.4 5. cry3a

The *cry3a* transcript was one of many *cry* genes predicted to be LWS1 enriched. This gene encodes a transcription factor involved in circadian regulation. This transcript was diffusely expressed in the retina of the 96 hpf embryo without particular localization to the photoreceptor layer (Supplementary Figure 3). T3 treatment did not appear to alter the expression domain of this gene (Supplementary Figure 3), and qPCR results also showed no significant difference between treatment groups (Supplementary Figure 3, *p* = 0.335).

Taken together, the outcomes of larval TH treatments provide only modest support for the second hypothesis that LWS cone transcripts are coordinately regulated by TH. These studies supported that *gngt2a* and *nrip1a* are upregulated by T3, although not in the same spatiotemporal manner as *lws1* [9] while *vax1*, *vax2*, and *cry3a* are unaffected, and *si:busm1-57f23.1* appears downregulated in the retina by T3. Further, *gngt2b* is downregulated by T3, again not in the same spatiotemporal manner as *lws2* [9], while *nr2f2* is unaffected. The LWS cone population therefore appears to display considerable heterogeneity in transcriptional response to TH.

## 4. Discussion

In this study we have probed the transcriptomes of long wavelength-sensitive cone photoreceptors of the zebrafish to advance our understanding of cone subtypes toward applications related to retinal development, function, and disease. The main findings of our study are as follows: LWS1 and LWS2 cones differ transcriptionally beyond opsin expression, and these differences include transcripts involved in photoreceptor function and development. Further, these cone subtypes show within-type heterogeneity. We also found that some of these transcriptional differences may be regulated by exogenous thyroid hormone, but in a manner that appears distinct from TH regulation of *lws1* vs. *lws2*.

### 4.1. LWS1 and LWS2 cones differ at the transcriptional level beyond opsin expression

In total, the bulk RNA-Seq results showed 95 transcripts enriched in LWS1 cones and 186 transcripts enriched in LWS2 cones, a finding that supports our first hypothesis that these cone subtypes differ beyond the level of opsin expression. To our knowledge, any such distinctions in the human LWS vs. MWS cone populations, other than opsin expression, have not been noted, although this is largely due to the challenges of detecting *LWS*-expressing vs. *MWS*-expressing cones within a dataset (Lukowski et al., 2019). The tandemly-replicated *LWS/MWS* opsin genes of primates display 98% homology at the level of transcript (Onishi et al., 2002) and are difficult to distinguish using standard RNA-Seq approaches. Interestingly, the scRNA-Seq approach of Peng et al. (2019) permitted this analysis for macaque cones, with the conclusion that these LWS and MWS cones are transcriptionally distinct only for the opsin genes, in contrast to our findings for the zebrafish.

Some of the transcripts enriched in zebrafish LWS1 vs. LWS2 cones suggest the possibility of further functional differences between the LWS cone subtypes. For example, the presence of the paralogous gamma transducin enriched in each cone subtype may reflect differences in phototransduction kinetics and/or recovery. However, to our knowledge such distinctions have not been experimentally tested. In addition, because multiple transcripts encoding factors involved in circadian rhythms were found to be enriched in LWS1 cones [*cry1bb*, *per3*, (Dataset 1) *cry1ba*, *aanat2*, *per2* (Dataset 4)], and no circadian related transcripts were enriched in LWS2 cones, LWS1 cones may have specialized functions in circadian rhythm. The majority of LWS1 cones are located within the ventral domain of the retina, exposed to direct sunlight, and therefore are in an ideal position to obtain circadian information (Takechi and Kawamura, 2005; Ogawa and Corbo, 2021). While the transcript tested using HCR *in situ* did not appear to be ventrally enriched, differences in sample collection timing may have been a factor (near lights-on for RNA-Seq, and midafternoon for adult whole retina collection), and retina-specific spatial expression data for the other circadian genes have not been reported in the literature.

Other differences in gene expression may give insight into the regulatory landscape of the retina. Multiple nuclear receptors and proteins that interact with nuclear receptors were predicted to be DE between LWS1 and LWS2 cones, including *nrip1a* and *nr2f2*. Given that ligands of nuclear hormone receptors (retinoic acid and TH) can regulate expression of *lws1* vs. *lws2* (Mitchell et al., 2015; Mackin et al., 2019), *nrip1a* and *nr2f2* may be considered candidates for participation in this regulation. The expression of *vax2* in LWS1 cones also proves interesting. *Vax2* is instrumental in regulating RA metabolism by altering the expression of RA-catabolizing and RA-synthesizing enzymes in developing mouse retina (Alfano et al., 2011). RA is known to be important in regulating cone opsin expression in multiple species (Prabhudesai et al., 2005; Roberts et al., 2005) including zebrafish, and specifically for regulating *lws1* vs. *lws2* (Mitchell et al., 2015), and RA receptors are known to heterodimerize with TH receptors (Berrodin et al., 1992). RA signaling takes place in ventral retina of juvenile zebrafish, spatially coinciding with a “transition zone” where LWS cones switch from *lws2* to *lws1* expression as the retina grows (Mitchell et al., 2015). Further, experimentally athyroid juvenile zebrafish display only a small ventral patch of *lws1* expression, which also spatially coincides with the RA signaling domain (Mackin et al., 2019). As *vax2* is known to be present in the zebrafish retina long before LWS cone development (French et al., 2009), this gene may play an upstream role in *lws* regulation by spatially tuning RA levels which, in turn, impart dorsoventral location information to developing LWS cones along with TH. While the presence of *vax2* in larval zebrafish retina is not surprising and likely is involved in the regulation of many genes, *vax2* expression in adult zebrafish was unexpected as *vax2* is not expressed in adult mouse retina (Barbieri et al., 1999). We speculate that *vax2* may be important in the maintenance of correct topography of cone subtypes in the adult zebrafish, perhaps even after regeneration (Stenkamp et al., 2021).

### 4.2. TH regulates several LWS1 and LWS2 enriched transcripts

We initially hypothesized that TH could be a master regulator of transcriptional differences between the LWS cone subtypes, LWS1 and LWS2. For this hypothesis to be true, DE genes would be coordinately regulated with *lws1* and *lws2* such that genes enriched in LWS1 cones would be upregulated by TH and genes enriched in LWS2 cones would be downregulated by TH. Our observations did not match this hypothesis. While *gngt2a* and *gngt2b* show some features of coordinated regulation with *lws1* and *lws2*, respectively, other transcripts such as *si:busm1* show the opposite–*si:busm1* was predicted to be enriched in LWS1 cones but appears downregulated by TH. *Nrip1a* is enriched in LWS1 cones and is upregulated by TH, similar to *lws1*, but its expression domain extends beyond the *lws1* domain and even beyond the photoreceptor layer. Other transcripts that are specifically enriched in LWS1 cones such as *vax1* and *vax2* display no transcriptional response to TH treatment. Therefore, TH is likely involved in regulation of transcripts other than *lws1* and *lws2*, but in a more complex manner than originally hypothesized, and perhaps independently of the *lws* opsin genes.

### 4.3. Transcriptional heterogeneity within the LWS cone population

Transcriptional heterogeneity among photoreceptor subtypes was recently described in detail for the zebrafish as the “partitioning” of expression of paralogs of photoreceptor components that emerged through whole genome duplications (Ogawa and Corbo, 2021). For example, the authors took note of partitioning of a paralog of the gamma subunit of transducin, *gngt2a*, within LWS1 and RH2-4 cones (Ogawa and Corbo, 2021), which express the most red-shifted members of the tandemly-replicated *lws* and *rh2* cone opsin gene arrays, respectively (Chinen et al., 2003). While our studies are largely consistent with this concept, the patterns of expression of the *gngt2* paralogs in cone subtypes appear more nuanced. Some LWS2 cones express *gngt2b*, but some express *gngt2a* or possibly both paralogs. Expression of *gngt2a* in adult retina is not limited to the ventral domain of LWS1 cones. Similarly, some but not all LWS1 cones express *vax1*. Some but not all LWS2 cones express *nr2f2*. For each of these LWS cone subtypes, there appears to be a great deal of transcriptional heterogeneity across the expanse of the retina.

Further heterogeneity is revealed within cone responses to treatment with exogenous TH–some but not all of these genes can be regulated by TH. Based on these observations, it appears that TH may be regulating these genes not because it is particularly regulating the entirety of LWS1 and LWS2 cone phenotype but rather because TH is an important regulator of spatial dynamics of gene expression in the zebrafish retina. Recent work in mouse supports this thyroid hormone-mediated spatial gradient regulation. It was found that *tr*β*2*, a TH receptor shared by mice and zebrafish, can control the expression and chromatin state of multiple cone genes that are expressed in a dorsoventral gradient in the mouse retina (Aramaki et al., 2022). This influential study also reveals heterogeneity within populations of mouse cones that express both *sws1* and *mws* opsins, in support of the concept that not all cones expressing a particular opsin are identical (Aramaki et al., 2022). The present study extends this concept to the *lws1*- expressing and *lws2*-expressing cone populations of the zebrafish. We aim to identify the TH receptor(s) that mediate the effects of TH in the zebrafish, as well as determine whether any are directly interacting with elements on the regulated gene.

While our approach was able to expand our understanding of LWS1 and LWS2 cone biology, we were limited in our ability to detect all of the potential transcriptional variability in these photoreceptors due to the limitations of sensitivity of bulk and scRNA-Seq. The bulk RNA-Seq results were further restricted by the effectiveness of the cell sorting procedure, and by the presence of proteasome-related transcripts in the LWS2 cone samples. Therefore we leveraged the bulk RNA-Seq datasets with information derived from a scRNA-Seq approach. We evaluated eight transcripts for their spatial patterning and TH responses. It is possible that many of the other differentially expressed genes may be regulated by TH. Further, these evaluations were done at the transcript level and do not reveal protein expression and/or localization, which would add another layer of support to the hypothesis that LWS1 and LWS2 cones are functionally distinct beyond opsin expression. In the future, we plan to perform single cell RNA-Seq on control and TH treated retinas to determine widespread effects of TH on the retinal cell transcriptomes. Due to the similarity of the LWS1 and LWS2 cone transcriptomes, LWS cone studies might also benefit from a manual photoreceptor collection technique, as in Angueyra et al. (2023), in order to increase the signal-to-noise ratio in the transcriptome data and reveal more potentially subtle gene expression differences.

Our results build upon our previous studies that showed *lws1* and *lws2* are regulated by TH (Mackin et al., 2019) by suggesting that multiple genes within the cones expressing these tandemly replicated opsins are non-stochastically regulated, and that these genes may also be regulated by TH. Further, our findings add to the growing literature that shows TH is a major regulator of spatial patterning in the retina and that its role is conserved across multiple species.

## Data availability statement

The datasets presented in this study can be found in the Gene Expression Omnibus (GEO) repository, accession numbers GSE232902 and GSE234661.

## Ethics statement

The animal study was reviewed and approved by the Institutional Animal Care and Use Committee of University of Idaho and Institutional Animal Care and Use Committee of University of Houston.

## Author contributions

AF, CS, ME, and AD performed the experiments. All authors analyzed the data, conceived the project, and wrote the manuscript.

## Footnotes

1
http://www.bioinformatics.babraham.ac.uk/projects/fastqc/

2
https://www.R-project.org/v.3.5.0

3
https://www.ncbi.nlm.nih.gov/geo/query/acc.cgi?acc=GSE232902

4
https://www.ncbi.nlm.nih.gov/geo/query/acc.cgi?acc=GSE234661
