# Supplementary material for: Long wavelength-sensing cones of zebrafish retina exhibit multiple layers of transcriptional heterogeneity
Source: Front Cell Neurosci. 2023 Jul 14;17:1214084. doi: 10.3389/fncel.2023.1214084 (PMC10382231; doi:10.3389/fncel.2023.1214084)
Supplement: Supplementary file 5 [file Presentation_1.PDF]

Supplemental Information for Farre et al., Long wavelength-sensing cones of zebrafish retina exhibit multiple layers of transcriptional heterogeneity

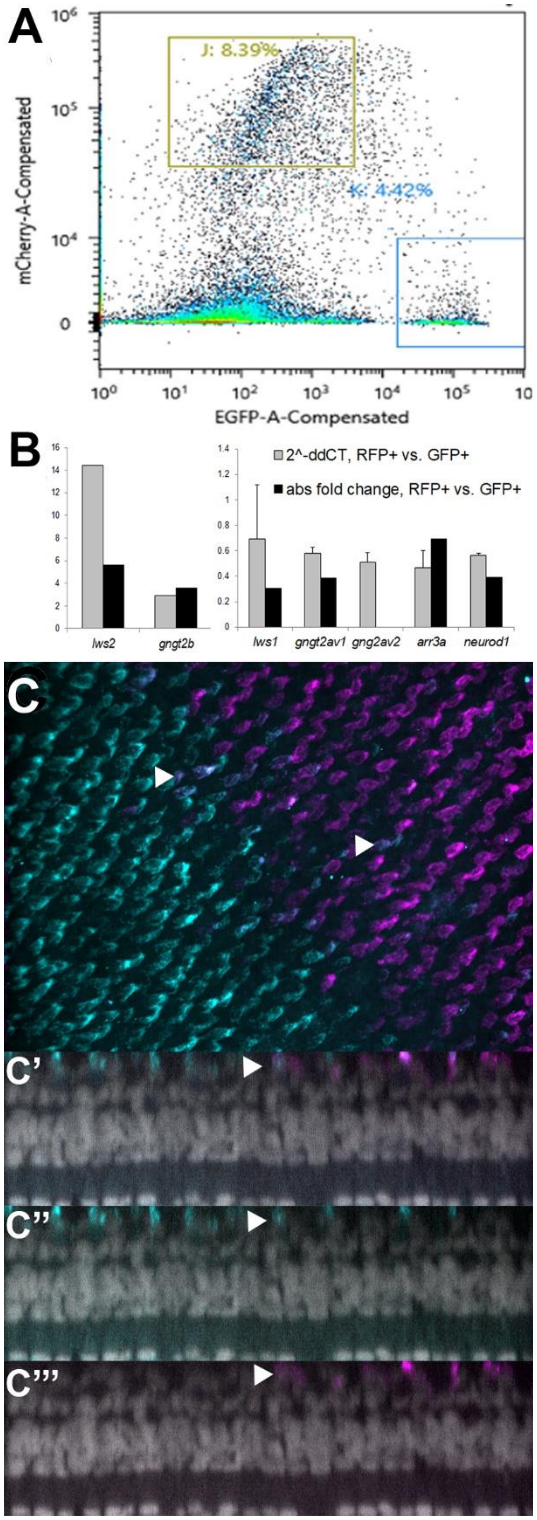

Supplemental Figure S1: qPCR validation of selected transcripts DE in LWS1 vs. LWS2 cones and evidence of some coexpression of *lws1* and *lws2* within LWS cones. A) Representative (100,000 sorted events) sorting report for an *lws:PAC(H)* sample used for validation (referred to as "Sort #2" in text; results from "Sort #1" were reported in [1]; red fluorescence intensity vs green fluorescence intensity. Gating strategy (boxes labeled J and K) for this sort resulted in the sorting percentages of events indicated. B) Column graphs of qPCR results (2^ddCT; gray columns) and RNA-Seq fold change (black columns) for selected transcripts DE in LWS2 cones (left graph; increased transcript abundance in RFP+ vs. GFP+ cones) and in LWS1 cones (right graph; decreased transcript abundance in RFP+ vs. GFP+ cones). C) Multiplex fluorescence HCR in situ hybridization for *lws1* (magenta) and *lws2* (cyan) in a region of whole mounted retina displaying cones that express both transcripts (arrows). C'-C''') Resliced orthogonal projections of region in C showing all three color channels (DAPI, gray; *lws1*, magenta; *lws2*, cyan) (C'), DAPI and *lws2* only (C''), DAPI and *lws1* only (C''').

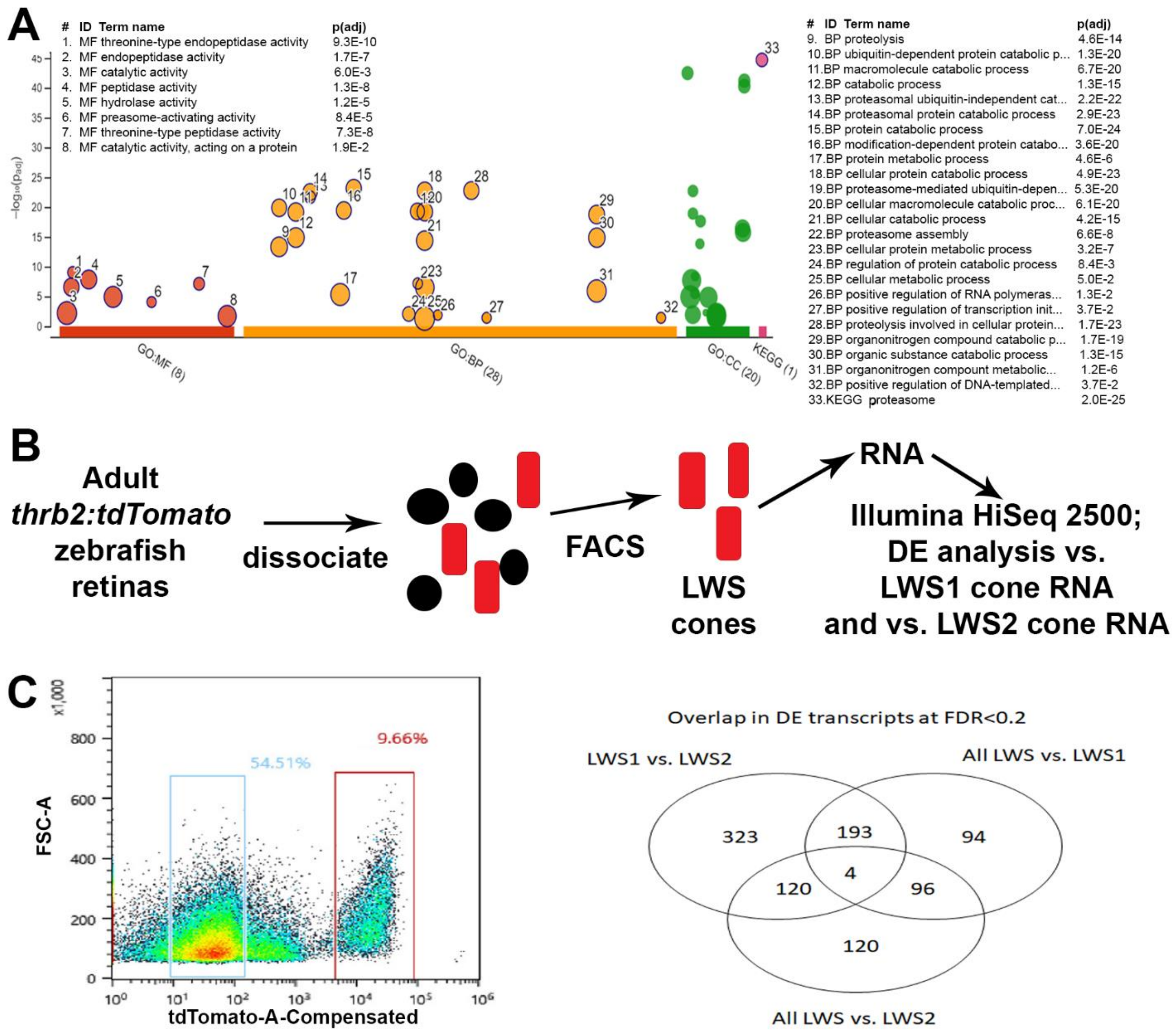

Supplemental Figure S2: A) Gene Ontology (GO) analysis depicting GO categories overrepresented in the list of DE genes enriched in LWS2 (vs. LWS1) cones. MF, molecular function; BP, biological process; CC, cellular component; KEGG, Kyoto encyclopedia of genes and genomes pathways. GO categories related to the proteasome dominate this analysis. B) Schematic of dissociation and sequencing workflow to obtain samples of LWS cones. C) Representative (100,000 sorted events) sorting report for an *thrb2:tdTomato* sample used in the study; red fluorescence intensity vs forward scatter. Gating strategy (red box) for this sort resulted in the sorting percentages of events indicated. D) Venn diagram of genes DE in LWS1 vs. LWS2 cones (from sort of *lws:PAC(H)* retinas), all LWS cones (from sort of *thrb2:tdTomato* retinas) vs. LWS1 cones (from sort of *lws:PAC(H)* retinas, and all LWS cones (from sort of *thrb2:tdTomato* retinas) vs. LWS2 cones (from sort of *lws:PAC(H)*). Approach identified transcripts DE in each LWS cone subtype as well as those common to both. Individual lists of transcripts within the overlapping regions of Venn diagram representing genes indicated as DE

in LWS2 cones (193 transcripts), and DE in LWS1 cones (120 transcripts) are provided in Dataset 3.

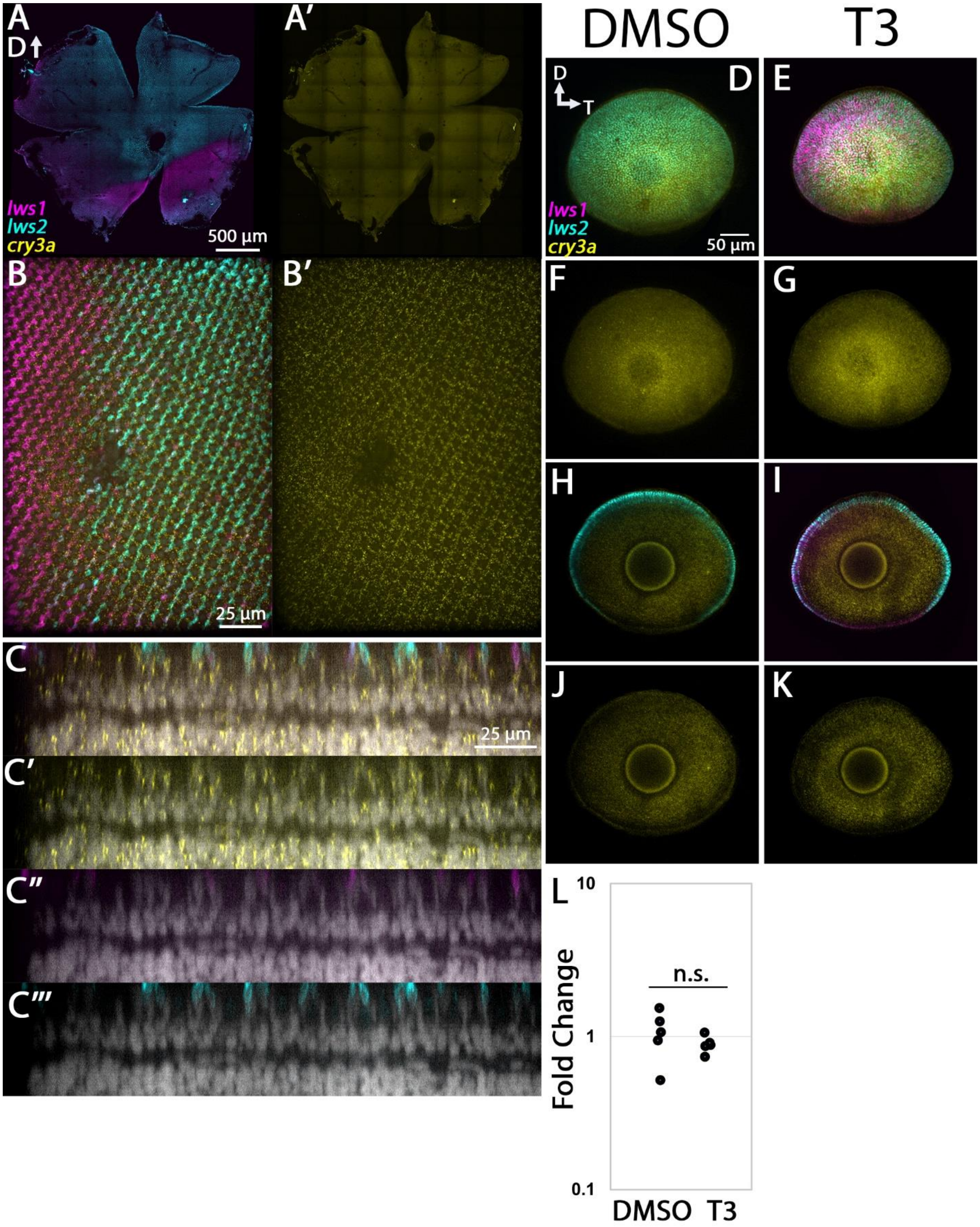

Supplemental Figure S3: Expression of *cry3a* in adult zebrafish retina (A-C), and in control (DMSO) and TH-treated (T3) larval zebrafish (D-K). A) Expression of *lws1* (magenta) and *lws2* (cyan) in a representative whole retina. A') *cry3a* expression (yellow) in the same preparation showing pan-retinal expression. B) 40x image of *lws1*, *lws2*, and *cry3a* expression in a region of *lws1* to *lws2* transition. B') 40x image of *cry3a* alone. C-C''') Resliced orthogonal projections of B. C) All imaging channels merged. C') DAPI and *cry3a*. C'') DAPI and *lws1.* C''') DAPI and *lws2.* Sample size (adults) =2. D-G) Projections of representative whole, imaged larval eyes. Note reduced expression domain of *lws2* (cyan), and expanded expression domain of *lws1* (magenta) but *cry3a* expression (yellow) appears unchanged in T3-treated (E, G) vs. controls (D, F). H-K) Single z slices obtained from the same preparations. L) qPCR quantification of *cry3a* transcript abundance in pooled samples of whole larvae, n=5 biological replicates per condition, p= 0.335. D = dorsal, T = temporal.

Supplemental Table S1: Primers used for qPCR.

**gene**	**forward primer**	**reverse primer**
beta actin	GTACCACCAGACAATACAGT	CTTCTTGGGTATGGAATCTTGC
gngt2a	GTGACCTGTTGCCTCCATCG	TTTAGAGACAGGCTCTCTGGT
gngt2b	ATCCACAGTCAGGATGGCTCG	TCGGCAGATAAACCCTCCAC
nrip1a	TACGAGCCTCTCCGACTCTT	GACAGCCCTGTTCGGGTG
nr2f2	ACACAGTCAACCCCGACGAACC	TTTGTCCCCGCAAACCACGC
vax1	TCTGCAGCAAACCCCTCTAC	TCGTACCCTGTTCGTCCTTC
vax2	AGAGACGCCAAGGGCACTAT	GAAACCACACTTTCACCTGTGTC
si:busm1	AGGCGGTAGTTGTAGCAAGAAA	TTGCTCTGGGCTTGCTGTTA
cry3a	ATCATTGGCGTCCACTACCC	GGAGGCCAGAAGTCCAAGTC

1. Sun, C., D.M. Mitchell, and D.L. Stenkamp, *Isolation of photoreceptors from mature, developing, and regenerated zebrafish retinas, and of microglia/macrophages from regenerating zebrafish retinas.* Exp Eye Res, 2018. **177**: p. 130-144.
